# Rational Design and Synthesis of Naphthalene Diimide Linked Bis-Naphthalimides as DNA Interactive Agents

**DOI:** 10.3389/fchem.2021.630357

**Published:** 2021-03-10

**Authors:** M. Shaheer Malik, Syed Farooq Adil, Ziad Moussa, Hatem M. Altass, Ismail I. Althagafi, Moataz Morad, Mohammad Azam Ansari, Qazi Mohammad Sajid Jamal, Rami J. Obaid, Abdulrahman A. Al-Warthan, Thokhir B. Shaik, Saleh A. Ahmed

**Affiliations:** ^1^ Department of Chemistry, Faculty of Applied Sciences, Umm Al-Qura University, Makkah, Saudi Arabia; ^2^ Department of Chemistry, College of Science, King Saud University, Riyadh, Saudi Arabia; ^3^ Department of Chemistry, College of Science, United Arab Emirates University, Al Ain, Abu Dhabi, United Arab Emirates; ^4^ Department of Epidemic Disease Research, Institute for Research & Medical Consultations (IRMC), Imam Abdulrahman Bin Faisal University, Dammam, 31441, Saudi Arabia; ^5^ Department of Health Informatics, College of Public Health and Health Informatics, Qassim University, Al Bukayriyah, Saudi Arabia; ^6^ Research on Advanced BioMedical Solutions Pvt Ltd, KPHB, Hyderabad, 500071, India; ^7^ Department of Chemistry, Faculty of Science, Assiut University, 71516, Assiut, Egypt; ^8^ Research Laboratories Unit, Faculty of Applied Science, Umm Al-Qura University, 21955, Makkah, Saudi Arabia

**Keywords:** bis-naphthalimides, naphthalene diimide, molecular modeling, DNA interactive, anticancer agents

## Abstract

A molecular modeling assisted rational design and synthesis of naphthalene diimide linked bis-naphthalimides as potential DNA interactive agents is described. Chemical templates incorporating naphthalene diimide as a linker in bis-naphthalimide motif were subjected to molecular docking analysis at specific intercalation and telomeric DNA G-quadruplex sites. Excellent results were obtained, which were better than the standards. A short and convenient synthetic route was employed to access these hybrids experimentally, followed by evaluation of their ability to cause thermal denaturation of DNA and cytotoxic properties along with ADME predictions. The obtained results provided useful insights and two potential molecules were identified for further development.

## Introduction

**FIGURE 1:**
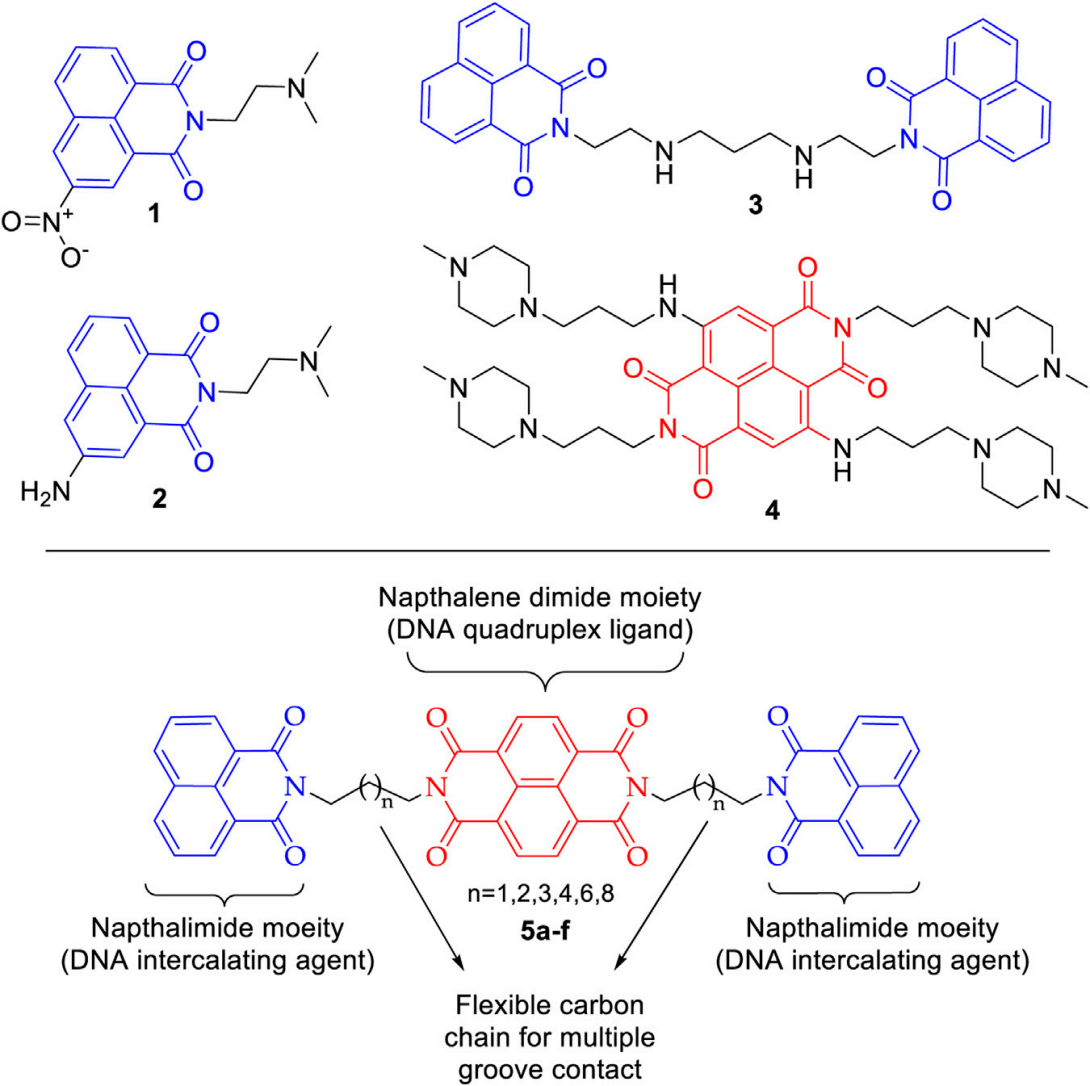
Design of naphthalene diimide linked bis-naphthalimides as DNA interactive agents.

DNA, the carrier of genetic information, is a prominent target in anticancer drug discovery programs. The drugs interact with the DNA and its associated proteins in varied manners resulting in cessation of the unbridled cell growth in cancer (Hurley, 2002; Izbicka and Tolcher, 2004; Delgado et al., 2018). Many DNA targeting drugs are currently in clinical use for treatment of different forms of cancer. Intercalation in DNA base pairs accompanied by interfering with DNA-protein interaction is one of the many mechanisms through which DNA interactive compounds exert cytotoxicity (Mukherjee and Sasikala, 2013). 1,8-Naphthalimide derivatives have emerged as important DNA intercalating class of compounds (Banerjee et al., 2013; Chen et al., 2018; Tomczyk and Walczak, 2018). Mitonafide (**1**) and amonafide (**2**) exhibited good DNA intercalation properties and were taken up for clinical studies with limited success (Casado et al., 1996; Allen and Lundberg, 2011). To further improve the DNA binding ability and the overall therapeutic profile of naphthalimides, the concept of bifunctional intercalating group was introduced. The designed bis-naphthalimides exhibited enhanced DNA binding properties and are integral area of research in DNA intercalators (Banerjee et al., 2013; Tandon et al., 2017). One such compound is elinafide (LU79553) (**3**), which is a lead molecule in the bis-naphthalimide class of compounds. Elinafide (**3**) exhibited encouraging antitumor activity in phase I clinical trial, though some cumulative toxicity issues were observed (Figure 1) (Villalona-Calero et al., 2001). On the other hand, in recent decades G-quadruplex DNA has emerged as an exciting target in cancer research (Hänsel-Hertsch et al., 2017). The G-quadruplex arrangement of 3′ end of human telomere inhibits the telomerase activity and can be exploited in the selective inhibition of cancer cell. For this purpose, small molecules that stabilize the telomeric end into a G-quadruplex structure are being developed (Monchaud and Teulade-Fichou, 2008). Naphthalene diimides (NDIs) scaffold is increasingly being harnessed as a suitable moiety in the design of quadruplex binding ligands because of its large planar surface (Ohnmacht et al., 2015; Pirota et al., 2019). In an interesting report, the NDI with *N*-methyl-piperazine side chain BMSG-SH-3 (**4**) bound exclusively to the 3′ surface of the DNA quadruplex, thus providing impetus to the design of new NDIs based molecules (Collie et al., 2012; Hampel et al., 2013). In addition to medicinal application, the NDIs have potential applications in molecular sensors, organic optoelectronics, photovoltaic cells, and flexible displays. These varied applications of NDIs stem from their characteristic high electron affinity, charge carrier capability, and profound oxidative as well as thermal stabilities (Kobaisi et al., 2016).

Cancer, being a disease of our own cells, is lethal with high rate of incidence and mortality. Extensive anticancer drug development programs are underway to develop newer drugs with enhanced efficacy and safety. The importance of bis-naphthalimides and naphthalene diimides in the design of new DNA interactive agents is well established with considerable success. Here, we envisaged chemical templates (**5a–f**) in which NDI motif is harnessed as a linker in a bis-naphthalimide scaffold. Further, to enhance the affinity of this template toward DNA intercalation and DNA quadruplex, the flexibility is regulated by six different carbon chain spacers. This could help in multiple groove contacts with DNA base pairs. The naphthalene diimide linked bis-naphthalimide templates were subjected to molecular docking to understand their DNA intercalating and telomeric G-quadruplex binding potential. This was followed by chemical synthesis of these compounds and *in vitro* evaluation of their DNA thermal denaturation property and cytotoxic potency against selected cancer cell lines along with ADME analysis.

## Materials and Methods

### Molecular Modeling

#### Chemical Templates Preparation

The chemical structures of the selected compounds were drawn using ChemDraw 19.1.0.8. Using chemical canonical SMILES IDs of the selected compounds, the 3D structures were obtained by CORINA classic 3D structure generator server (https://www.mn-am.com/online_demos/corina_demo). Discovery Studio Visualizer, 2019 was used to implement CHARMM force field to complete energy minimization process for the generated 3D structures of selected compounds (Vanommeslaeghe et al., 2010).

#### DNA Molecules Preparation

The 3D structures of DNA molecules (PDB:1CX3 and PDB:3SC8) were downloaded from Protein Data Bank (PDB) (www.rcsb.org). Further HETATOM and water molecules were removed from the native PDB 3D structure files. For energy minimization CHARMM force field was applied using Discovery Studio Visualizer, 2019.

#### Computational Docking Studies

*In silico* docking was performed between compounds (**5a–f**) and DNA molecules (PDB:1CX3 and PDB:3SC8) using AutoDock 4.2 software. AutoDock runs Lamarckian Genetic Algorithm (LGA) and empirical binding free energy function as a scoring function for the ligand-receptor interaction (Morris et al., 1998; Morris et al., 2009). The docking was executed implementing the default AutoDock parameters; however, to cover the maximum area 60 × 60 × 60 Å grid box was used and the grid center point coordinates X, Y, and Z were set as 0.739, −0.12, −0.024 for PDB:1CX3 and −12.145, −15.576, 4.968 for PDB:3SC8, respectively, with the default value of grid points spacing 0.375 Å. After successful completion of docking steps, the results were analyzed and the graphics generation was performed using Discovery Studio Visualizer, 2019.

### Chemical Synthesis

Reactions were monitored by thin layer chromatography (silica gel glass plates containing 60 F-254) and visualization was achieved by UV light or iodine indicator. Infrared (IR) spectra were recorded on Perkin-Elmer model 683 or 1,310 spectrometers with sodium chloride optics. ^1^H NMR spectra were recorded on Gemini (200 MHz) (Varian Inc., Palo Alto, CA, United States) and chemical shifts (d) were reported in ppm, downfield from internal TMS standard. ESI spectra were recorded on Micromass Quattro LC using ESIþ software with capillary voltage of 3.98 kV and ESI mode positive ion trap detector. Elemental analyses were performed on an elemental analyzer (Model: VARIO EL, Elementar, Hanau, Germany). Starting materials and reagents were purchased from Sigma-Aldrich and Alfa-Aesar.

#### General Procedure for Synthesis of *N*-Alkyl Azido Naphthalimide Derivatives **9a–f**

A mixture of naphthalimide **6** (1 equiv) and dibromoalkanes **7a–f** (3 equiv) was dissolved in acetonitrile, K_2_CO_3_ (5 equiv) was added, and the resulting solution was refluxed at 80°C for about 3–4 h. The reaction was monitored using TLC and after completion of reaction, the mixture was filtered through sintered funnel. Then the resulting filtrate was concentrated in vacuo and the residue was washed with petroleum ether to remove the unreacted dibromoalkane providing the crude product **8a–f**, which was used for azidation reaction. A mixture of *N*-3-bromopropyl naphthalimide **8a–f** (1 equiv) and sodium azide (3 equiv) was dissolved in DMF and the resulting mixture was stirred at room temperature. The reaction was monitored using TLC and after the completion of reaction, the reaction mixture was added to ice-cold water and the resulting solution was extracted with ethyl acetate. Then the resulting filtrate was concentrated in vacuo and the products **9a–f** were isolated using column chromatography.

#### 2-(3-Azidopropyl)-2,3-dihydro-1H-benzo[de]isoquinoline-1,3-dione (**9a**)

It is prepared by following the general procedure of **9a–f** from naphthalimide **6** (200 mg, 1.015 mmol), 1,3 dibromopropane **7a** (612 mg, 3.045 mmol), and K_2_CO_3_ (700 mg, 5.076 mmol) in first step to provide **8a**. This is followed by azidation of **8a** (200 mg, 0.628 mmol) using sodium azide (122 mg, 1.886 mmol) to afford 160 mg of **9a** as gummy solid. Yield: 94%; IR (KBr): ν 2,090, 1,685, 1,662, 1,155 cm^−1^. ^1^H NMR (200 MHz, CDCl_3_): δ ppm 1.58–2.12 (m, 4H), 3.28–3.52 (m, 2H), 7.62–8.33 (m, 6H). MS (ESI): m/z 280 (M^+^). Anal. Calcd for C_15_H_12_N_4_O_2_: C, 64.28; H, 4.32; N, 19.99 Found: C, 64.02; H, 4.28; N, 19.56%.

#### 2-(4-Azidobutyl)-2,3-dihydro-1H-benzo[de]isoquinoline-1,3-dione (**9b**)

It is prepared by following the general procedure of **9a–f** from naphthalimide **6** (200 mg, 1.015 mmol), 1,4-dibromobutane **7b** (654 mg, 3.045 mmol), and K_2_CO_3_ (700 mg, 5.076 mmol) in first step to provide **8b**. This is followed by azidation of **8b** (200 mg, 0.602 mmol) using sodium azide (117 mg, 1.807 mmol) to afford 165 mg of **9b** as gummy solid. Yield: 93%; IR (KBr): ν 2,103, 1,682, 1,659, 1,162 cm^−1^.^1^H NMR (200 MHz, CDCl_3_): δ ppm 1.23–2.03 (m, 6H), 3.28–3.48 (m, 2H), 7.57–8.21 (m, 6H). MS (ESI): m/z 294 (M^+^). Anal. Calcd for C_16_H_14_N_4_O_2_: C, 65.30; H, 4.79; N, 19.04 Found: C, 65.27; H, 4.68; N, 18.89%.

#### 2-(5-Azidopentyl)-2,3-dihydro-1H-benzo[de]isoquinoline-1,3-dione (**9c**)

It is prepared by following the general procedure of **9a–f** from naphthalimide **6** (200 mg, 1.015 mmol), 1,5-dibromopentane **7c** (697 mg, 3.045 mmol), and K_2_CO_3_ (700 mg, 5.076 mmol) in first step to provide **8c**. This is followed by azidation of **8c** (200 mg, 0.578 mmol) using sodium azide (112 mg, 1.734 mmol) to afford 162 mg of **9c** as gummy solid. Yield: 91%; IR (KBr): ν 2,085, 1,678, 1,659, 1,162 cm^−1^. ^1^H NMR (200 MHz, CDCl_3_): δ ppm 1.16–2.11 (m, 8H), 3.19–3.42 (m, 2H), 7.37–8.19 (m, 6H). MS (ESI): m/z 308 (M^+^). Anal. Calcd for C_17_H_16_N_4_O_2_: C, 66.22; H, 5.23; N, 18.17 Found: C, 65.98; H, 5.06; N, 18.20%.

#### 2-(6-Azidohexyl)-2,3-dihydro-1H-benzo[de]isoquinoline-1,3-dione (**9d**)

It is prepared by following the general procedure of **9a–f** from naphthalimide **6** (200 mg, 1.015 mmol), 1,6-dibromohexane **7d** (739 mg, 3.045 mmol), and K_2_CO_3_ (700 mg, 5.076 mmol) in first step to provide **8d**. This is followed by azidation of **8d** (200 mg, 0.555 mmol) using sodium azide (108 mg, 1.66 mmol) to afford 170 mg of **9d** as gummy solid. Yield: 94%; IR (KBr): ν 2,095, 1,689, 1,665, 1,162 cm^−1^. ^1^H NMR (200 MHz, CDCl_3_): δ ppm 1.21–2.19 (m, 10H), 3.21–3.38 (m, 2H), 7.29–8.21 (m, 6H). MS (ESI): m/z 322 (M^+^). Anal. Calcd for C_18_H_18_N_4_O_2_: C, 67.07; H, 5.63; N, 17.38 Found: C, 67.01; H, 5.56; N, 17.22%.

#### 2-(8-Azidooctyl)-2,3-dihydro-1H-benzo[de]isoquinoline-1,3-dione (**9e**)

It is prepared by following the general procedure of **9a–f** from naphthalimide **6** (200 mg, 1.015 mmol), 1,8-dibromooctane **7e** (828 mg, 3.045 mmol), and K_2_CO_3_ (700 mg, 5.076 mmol) in first step to provide **8e**. This is followed by azidation of **8e** (200 mg, 0.515 mmol) using sodium azide (100 mg, 1.546 mmol) to afford 168 mg of **9e** as gummy solid. Yield: 94%; IR (KBr): ν 2,102, 1,688, 1,663, 1,156 cm^−1^. ^1^H NMR (200 MHz, CDCl_3_): δ ppm 1.29–2.02 (m, 14H), 3.19–3.28 (m, 2H), 7.33–8.29 (m, 6H). MS (ESI): m/z 350 (M^+^). Anal. Calcd for C_20_H_22_N_4_O_2_: C, 68.55; H, 6.33; N, 15.99 Found: C, 68.50; H, 6.25; N, 15.92%.

#### 2-(10-Azidodecyl)-2,3-dihydro-1H-benzo[de]isoquinoline-1,3-dione (**9f**)

It is prepared by following the general procedure of **9a–f** from naphthalimide **6** (200 mg, 1.015 mmol), 1,10-dibromodecane **7f** (913 mg, 3.045 mmol), and K_2_CO_3_ (700 mg, 5.076 mmol) in first step to provide **8f**. This is followed by azidation of **8f** (200 mg, 0.480 mmol) using sodium azide (93 mg, 1.442 mmol) to afford 166 mg of **9f** as gummy solid. Yield: 92%; IR (KBr): ν 2095, 1,689, 1,659, 1,152 cm^−1^. ^1^H NMR (200 MHz, CDCl_3_): δ ppm 1.21–2.12 (m, 18H), 3.15–3.29 (m, 2H), 7.31–8.32 (m, 6H). MS (ESI): m/z 378 (M^+^). Anal. Calcd for C_22_H_26_N_4_O_2_: C, 69.82; H, 6.92; N, 14.80 Found: C, 69.78; H, 6.88; N, 14.75%.

#### General Procedure for Synthesis of Naphthalene Diimide Linked Bis-Naphthalimides **5a–f**

A mixture of *N*-3-azidopropyl naphthalimide **9a–f** (1 equiv) and naphthalic dianhydride **10** (2 equiv) was dissolved in acetic acid. Sodium iodide (6 equiv) was added and the resulting mixture was stirred at reflux temperature for 12 h. After the reaction time, ethyl acetate was added and stirred for 10 min and the organic layer was washed with aq. NaHCO_3_ followed by aq. Na_2_S_2_O_3_ solution. The organic layer was then concentrated under reduced pressure to obtain crude product which was purified using preparative TLC using hexane:ethyl acetate as solvent system to afford desired compounds **5a–f** as white solid.

#### 2,7-Di[3-(1,3-dioxo-2,3-dihydro-1H-benzo[de]isoquinolin-2-yl)propyl]-1,2,3,6,7,8-hexahydrobenzo[lmn][3,8]phenanthroline-1,3,6,8-tetraone (**5a**)

It is prepared by following the general procedure of **5a–f** from *N*-3-azidopropyl naphthalimide **9a** (100 mg, 0.357 mmol), naphthalic dianhydride **10** (191 mg, 0.710 mmol), and NaI (321 mg, 2.142 mmol). 105 mg of product **5a** was obtained as white solid. Yield: 40%; mp 141°C; IR (KBr): ν 2,950, 1,696, 1,664, 1,160 cm^−1^. ^1^H NMR (200 MHz, CDCl_3_): δ ppm 2.23–2.45 (m, 4H), 3.38–3.61 (m, 8H), 7.62–7.91 (m, 4H), 8.11–8.31 (m, 8H), 8.58 (d, 4H). ^13^C NMR (75 MHz, CDCl_3_): δ ppm 161.1, 136.3, 134.3, 131.8, 130.7, 127.49, 125.3, 125.1, 42.1, 37.4. MS (ESI): m/z 763 (M+23). Anal. Calcd. for C_44_H_28_N_4_O_8_: C, 71.35; H, 3.81; N, 7.56 Found: C, 71.30; H, 3.78; N, 7.54%.

#### 2,7-Di[4-(1,3-dioxo-2,3-dihydro-1H-benzo[de]isoquinolin-2-yl)butyl]-1,2,3,6,7,8-hexahydrobenzo[lmn][3,8]phenanthroline-1,3,6,8-tetraone (**5b**)

It is prepared by following the general procedure of **5a–f** from *N*-3-azidopropyl naphthalimide **9b** (100 mg, 0.340 mmol), naphthalic dianhydride **10** (182 mg, 0.680 mmol), and NaI (306 mg, 2.04 mmol). 94 mg of product **5b** was obtained as white solid, yield 36%; mp 151°C. IR (KBr): ν 2,948, 1,697, 1,666, 1,155 cm^−1^.^1^H NMR (200 MHz, CDCl_3_): δ ppm 1.42–1.85 (m, 8H), 3.37–3.63 (m, 8H), 7.61–7.93 (m, 4H), 8.12–8.29 (m, 8H), 8.58 (d, 4H). MS (ESI): m/z 768 (M^+^). Anal. Calcd for C_46_H_32_N_4_O_8_: C, 71.87; H, 4.20; N, 7.29 Found: C, 71.80; H, 4.16; N, 7.25%.

#### 2,7-Di[5-(1,3-dioxo-2,3-dihydro-1H-benzo[de]isoquinolin-2-yl)pentyl]-1,2,3,6,7,8-hexahydrobenzo[lmn][3,8]phenanthroline-1,3,6,8-tetraone (**5c**)

It is prepared by following the general procedure of **5a–f** from *N*-3-azidopropyl naphthalimide **9c** (100 mg, 0.308 mmol), naphthalic dianhydride **10** (173 mg, 0.649 mmol), and NaI (277 mg, 1.848 mmol). 100 mg of product **5c** was obtained as white solid, yield 39%; mp 156°C. IR (KBr): ν 2,954, 1700, 1,670, 1,060 cm^−1^. ^1^H NMR (200 MHz, CDCl_3_): δ ppm 1.44–1.88 (m, 12H), 3.36–3.69 (m, 8H), 7.59–7.82 (m, 4H), 8.11–8.31 (m, 8H), 8.58 (d, J = 7.38 Hz, 4H). ^13^C NMR (75 MHz, CDCl_3_): δ ppm 161.1, 136.3, 134.3, 131.8, 130.7, 127.49, 125.4, 125.1, 42.1, 28.9, 28.4, 27.6. MS (ESI): m/z 819 (M+23). Anal. Calcd for C_48_H_36_N_4_O_8_: C, 72.35; H, 4.55; N, 7.03 Found: C, 72.30; H, 4.51; N, 7.00%.

#### 2,7-Di[6-(1,3-dioxo-2,3-dihydro-1H-benzo[de]isoquinolin-2-yl)hexyl]-1,2,3,6,7,8-hexahydrobenzo[lmn][3,8]phenanthroline-1,3,6,8-tetraone (**5d**)

It is prepared by following the general procedure of **5a–f** from *N*-3-azidopropyl naphthalimide **9d** (100 mg, 0.289 mmol), naphthalic dianhydride **10** (155 mg, 0.579 mmol), and NaI (260 mg, 1.734 mmol). 94 mg of product **5d** was obtained as white solid, yield 37%; mp 162°C. IR (KBr): ν 2,948, 1,697, 1,666, 1,075 cm^−1^. ^1^H NMR (200 MHz, CDCl_3_): δ ppm 1.43–1.88 (m, 16H), 3.34–3.62 (m, 8H), 7.55–7.79 (m, 4H), 8.13–8.30 (m, 8H), 8.56 (d, 4H). MS (ESI): m/z 874 (M+23). Anal. Calcd for C_50_H_40_N_4_O_8_: C, 72.80; H, 4.89; N, 6.79 Found C, 72.80; H, 4.89; N, 6.79%.

#### 2,7-Di[8-(1,3-dioxo-2,3-dihydro-1H-benzo[de]isoquinolin-2-yl)octyl]-1,2,3,6,7,8-hexahydrobenzo[lmn][3,8]phenanthroline-1,3,6,8-tetraone (**5e**)

It is prepared by following the general procedure of **5a-f** from *N*-3-azidopropyl naphthalimide **9e** (100 mg, 0.285 mmol), naphthalic dianhydride **10** (153 mg, 0.571 mmol), and NaI (256 mg, 1.71 mmol). 95 mg of product **5e** was obtained as white solid, yield 38%; mp 172°C. IR (KBr): ν 2,948, 1706, 1,660, 1,068 cm^−1^. ^1^H NMR (200 MHz, CDCl_3_): δ ppm 1.41–1.87 (m, 24H), 3.39–3.60 (m, 8H), 7.63–7.84 (m, 4H), 8.10–8.30 (m, 8H), 8.58 (d, 4H). MS (ESI): m/z 880 (M+). Anal. Calcd for C_54_H_48_N_4_O_8_: C, 73.62; H, 5.49; N, 6.36 Found: C, 73.60; H, 5.44; N, 6.34%.

#### 2,7-Di[10-(1,3-dioxo-2,3-dihydro-1H-benzo[de]isoquinolin-2-yl)decyl]-1,2,3,6,7,8-hexahydrobenzo[lmn][3,8]phenanthroline-1,3,6,8-tetraone (**5f**)

It is prepared by following the general procedure of **5a–f** from *N*-3-azidopropyl naphthalimide **9f** (100 mg, 0.264 mmol), naphthalic dianhydride **10** (141 mg, 0.529 mmol), and NaI (238 mg, 1.584 mmol). 88 mg of product **5f** was obtained as white solid, yield 36%; mp 184°C. IR (KBr): ν 2,951, 1,694, 1,670, 1,074 cm^−1^. ^1^H NMR (200 MHz, CDCl_3_): δ ppm 1.43–1.98 (m, 32H), 3.38–3.61 (m, 8H), 7.62–7.82 (m, 4H), 8.11–8.31 (m, 8H), 8.58 (d, 4H). MS (ESI): m/z 960 (M+23). Anal. Calcd for C_58_H_56_N_4_O_8_: C, 74.34; H, 6.02; N, 5.98 Found: C, 74.34; H, 6.02; N, 5.98%.

### Biological Assays

#### Thermal Denaturation Studies

Compounds **5a–f** were subjected to thermal denaturation studies with duplex-form CT-DNA using reported method (Kamal et al., 2002). Working solutions in aqueous buffer (10 mM NaH_2_PO_4_/Na_2_HPO_4_, 1 mM Na_2_EDTA, pH 7.00 ± 0.01) containing CT-DNA (100 μM in phosphate) and the **5a–f** (20 μM) were prepared by addition of concentrated **5a–f** solutions in DMSO to obtain a fixed [**5a–f** ]/[DNA] molar ratio of 1:5. The DNA–**5a–f** solutions were incubated at 37°C for 0, 18, and 36 h prior to analysis. Samples were monitored at 260 nm using a BECKMAN COULTER DU 800 spectrophotometer fitted with high performance temperature controller, and heating was applied at 1°C min^−1^ in the 40–90°C range. DNA helix→coil transition temperature (*T*_m_) has been obtained from the maxima in the d(A260)/dT derivative plots. Results have been given as the mean ± standard deviation from three determinations and are corrected for the effects of DMSO cosolvent using a linear correction term. Drug-induced alterations in DNA melting behavior have been calculated by ∆*T*m =*T*m (DNA+ **5a–f**)-Tm (DNA alone), where the *T*m value for the [**5a–f**]-free CT-DNA is 69.2 ± 0.01°C.

#### Cytotoxicity Assay

*In vitro* anticancer screening assay was performed according to NCI procedures in triplicate. A panel of ten cancer lines belonging to seven different cancer types, prostrate (DU145, PC-3), colon (colo-205, HCT-15, 502713), breast (MCF-7), neuroblastoma (IMR-32), liver (Hep-2), ovary (OVCAR-5), and lung cancers (A549), was selected. The human tumor cell lines of the cancer screening panel were grown in RPMI 1640 medium containing 5% fetal bovine serum and 2 mM l-glutamine. For a typical screening experiment, cells were inoculated into 96-well microtiter plates in 100 μL at plating densities ranging from 5,000 to 40,000 cells/well depending on the doubling time of individual cell lines. After cell inoculation, the microtiter plates were incubated at 37°C, 5% CO_2_, 95% air, and 100% relative humidity for 24 h prior to addition of experimental drugs. Experimental drugs were solubilized in dimethyl sulfoxide at 400-fold the desired final maximum test concentration and stored frozen prior to use. Following drug addition, the plates were incubated for an additional 48 h at 37°C, 5% CO_2_, 95% air, and 100% relative humidity. For adherent cells, the assay was terminated by the addition of cold trichloro acetic acid (TCA). Sulforhodamine B (SRB) solution (100 μL) at 0.4% (w/v) in 1% acetic acid was added to each well, and plates are incubated for 10 min at room temperature. After staining, unbound dye is removed by washing five times with 1% acetic acid and the plates were air-dried. Bound stain was subsequently solubilized with 10 mM trizma base, and the absorbance was read on an automated plate reader at a wavelength of 515 run. Using the absorbance measurements [time zero, (Tz), control growth in DMSO without drug, (C), and test growth in the presence of drug at the different concentration levels (Ti)], the percentage growth was calculated at each of the drug concentrations levels.

### ADME Prediction

ADME profiling of compounds was carried out using computational approach. ADME properties of compounds **5a**–**5f** and standards, elinafide (**3**) and BMSG-SH-3 (**4**), were calculated using preADMET and swissADME predictor online programs (Lee et al., 2003; Daina et al., 2017). The properties like BBB, PPB, Caco2, P-gp interaction, and TPSA were analyzed to understand the ADME properties of these compounds.

## Results and Discussions

### Molecular Modeling

**FIGURE 2:**
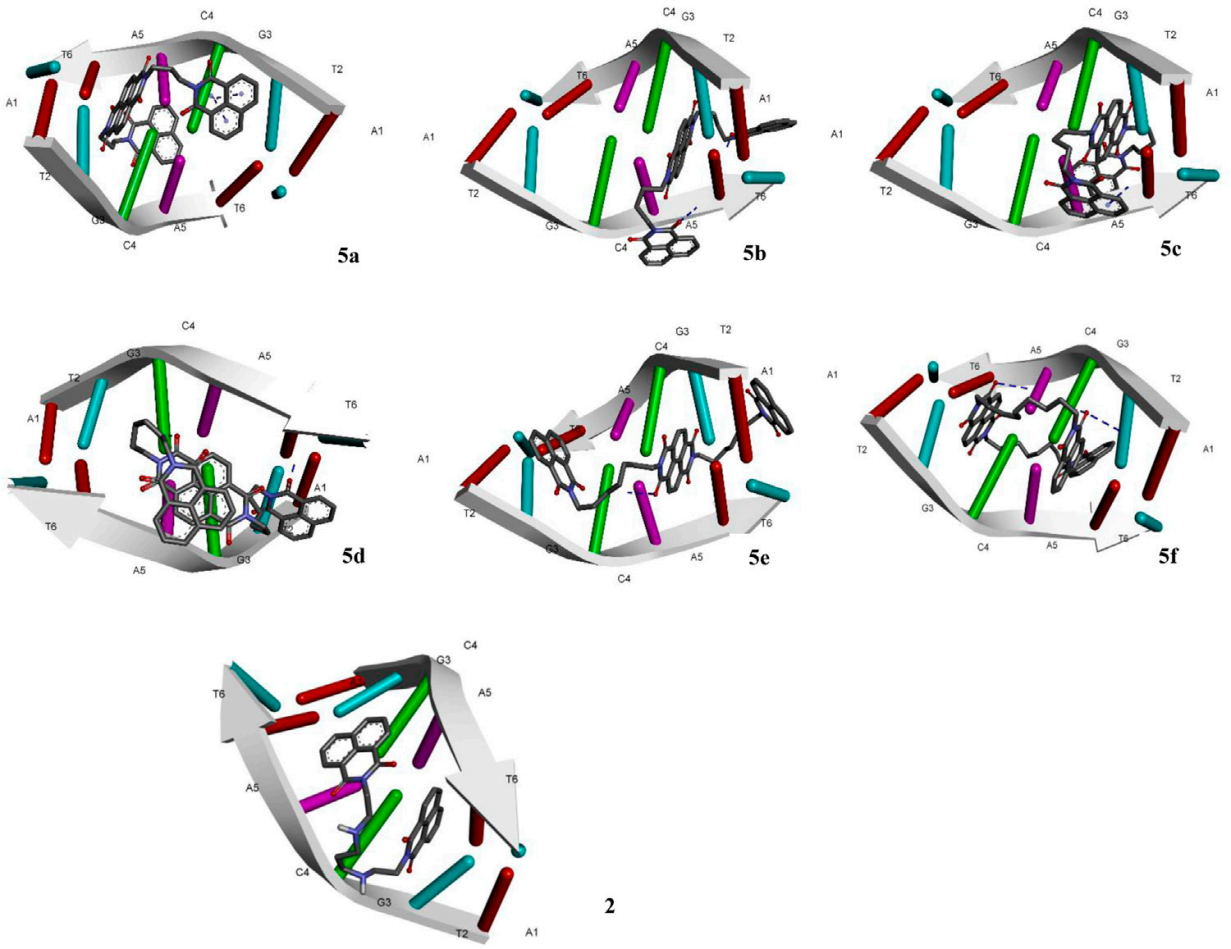
Molecular docking of compounds **5a**–**f** and standard elinafide **2** at d(atgcat)2 site of DNA revealing intercalation ability.

The designed chemical entities incorporating the DNA interacting bis-naphthalimides and naphthalene diimides moieties were subjected to DNA interaction analysis at molecular level using AutoDock software. First, the compounds **5a–f** were studied for their DNA intercalating ability at d(atgcat)2 site of DNA and compared with the standard **3** (Figure 2). The results revealed that the compounds exhibited excellent DNA intercalating property compared to the standard **3** (Table 1). The binding affinity of most of the compounds was better than **3** with compound **5a** exhibiting exceptional binding affinity score of −10.93 kcal/mol and inhibition constant of 9.69 nM. The interaction of **5a** with the DNA molecule resulted through the formation of three hydrogen bonds with bond lengths of 2.67–3.31 Å and other noncovalent interactions. In comparison the standard **3** exhibited binding affinity of −6.90 kcal/mol and inhibition constant of 8.73 uM with only single hydrogen bond formation. The compound **5b** interacted with the DNA site with two hydrogen bonds and a binding energy of −9.45 kcal/mol. Similarly, in case of the compounds **5c** and **5f** also the binding affinity was better than **3** with values of −9.69 and −9.01 kcal/mol resulting from one and three hydrogen bonds’ formation, respectively, with **5c** showing an inhibition constant of 79.37 nM. Comparatively, low binding affinities were observed in compounds **5d** and **5e** with formation of one and two hydrogen bonds and a resulting binding energy of −6.32 and −7.63 kcal/mol, respectively.

**TABLE 1:** Molecular docking analysis of some critical parameters of compounds **5a–f** and elinafide **3** at d(atgcat)2 site of DNA^a^.

S. No.	Compound	Binding affinity (Kcal/mol)	Inhibition constant	Hydrogen bonds	Hydrogen bonds lengths (Angstrom)	No. of hydrophobic interactions^b^	No. of pi-pi and pi-alkyl interactions^c^
1	Control **3**	−6.90	8.73 uM	A:DG3:O4’- :UNK1:C17	3.33	3	4
2	**5a**	−10.93	9.69 nM	A:DG3:H7 - :UNK1	2.86	2	4
				A:DG3:H7 - :UNK1	2.67		
				A:DG3:H7 - :UNK1	3.31		
3	**5b**	−9.45	117.53 nM	A:DA5:H7 - :UNK1:O56	2.52	—	3
				A:DA5:H3 - :UNK1:O14	1.90		
4	**5c**	−9.69	79.37 nM	A:DA5:H62 - :UNK1	2.65	3	3
5	**5d**	−6.32	23.35 uM	A:DG3:H22 - :UNK1:O35	1.97	4	2
				A:DA5:H3 - :UNK1:O60	1.96		
6	**5e**	−7.63	2.55 uM	A:DC4:H42 - :UNK1:O39	2.65	2	4
7	**5f**	−9.01	248.18 nM	A:DG3:H7 - :UNK1:O54	2.73	2	3
				A:DC4:H42 - :UNK1:O41	2.92		
				A:DT2:C6 - :UNK1:O54	3.35		

^a^ See Supplementary Material for more information.

^b,c^ Other noncovalent interactions between the ligands and the intercalation site of DNA.

In addition to the hydrogen bonding interactions, some other noncovalent interactions such as hydrophobic interactions and pi–pi and pi-alkyl interactions were also observed. The compound **5d** exhibited most hydrophobic interactions with the DNA helix, followed by compound **5c** that showed three interactions similar to standard **3**. Two hydrophobic interactions were seen in other compounds and compound **5b** exhibited no such interaction. Other noncovalent interactions (pi-pi and pi-alkyl) were also observed and compound **5a** as well as **5e** showed maximum of these interactions, similar to standard **3** (see Supplementary Material).

**FIGURE 3:**
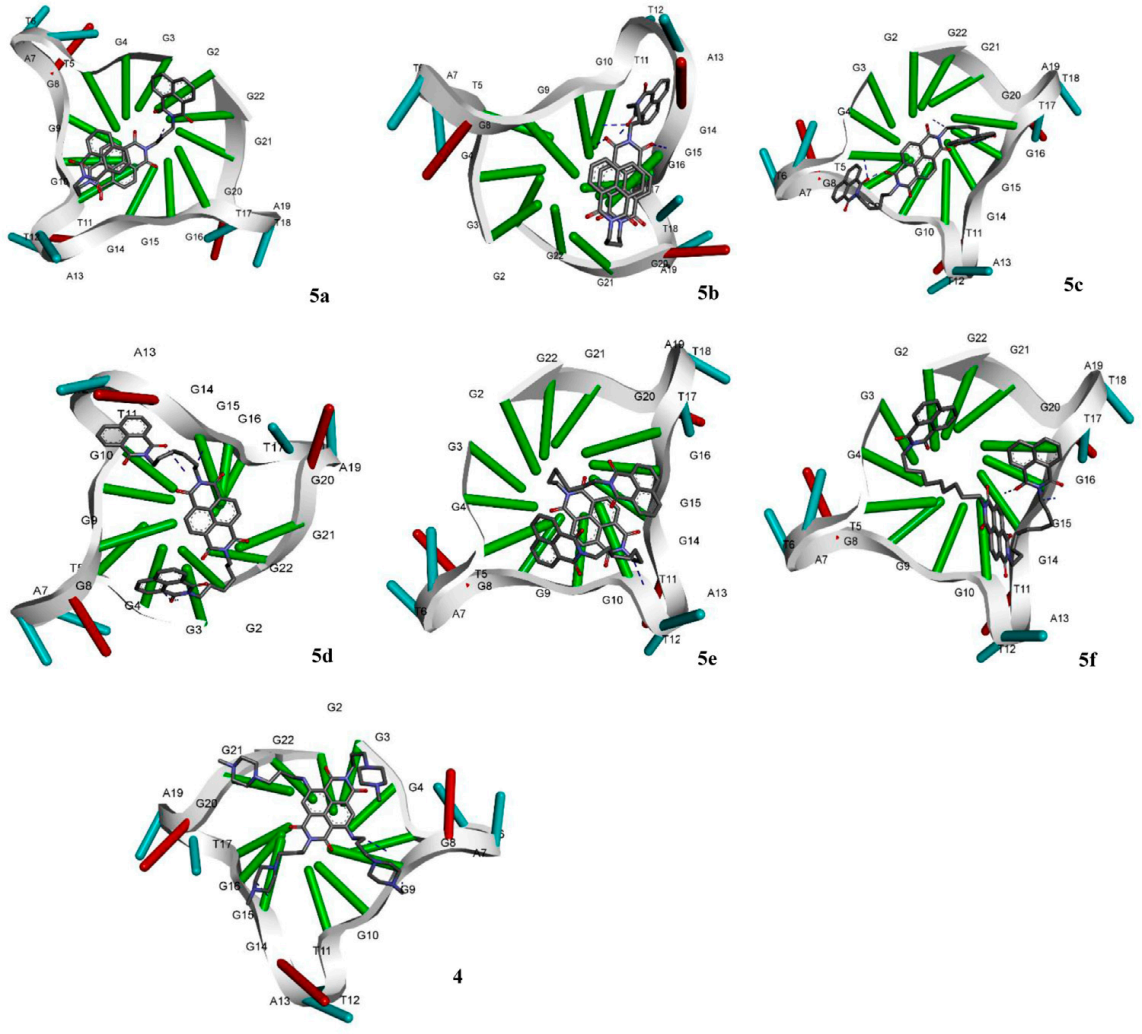
Molecular docking of compounds **5a**–**f** and standard BMSG-SH-3 (**4**) with telomeric G-quadruplex, a promising cancer target.

The molecular docking of the compounds **5a–f** for DNA intercalating ability exhibited excellent results and encouraged us to study their interaction with telomeric G-quadruplex, a promising cancer target. The tetrameric naphthalene diimide derivative BMSG-SH-3 (**4**) was used as a control (Figure 3). All the compounds **5a-f** showed binding affinity in the range of −4.15 to −7.82 kcal/mol, which were better than the standard **3** (−3.70 kcal/mol) as shown in Table 2. The compound **5b** was the best among the series with a binding affinity score of −7.82 kcal/mol resulting from formation of four hydrogen bonds with the nitrogen base pairs of quadruplex DNA and an inhibition constant of 1.86 uM. This was higher than three hydrogen bonds’ formation exhibited by the standard **3.** Similarly to **5b**, the compound **5d** also exhibited good binding with telomeric G-quadruplex via four hydrogen bonds’ formation with binding energy and inhibition constant scores of −7.47 kcal/mol and 3.34 uM, respectively. This was followed by compound **5c**, which exhibited a binding affinity of −6.42 kcal/mol with the formation of three hydrogen bonds with nitrogen base pairs. The binding energies of −5.33 and −4.81 kcal/mol were shown by compounds **5a** and **5e,** respectively, with one hydrogen bond’s formation. On the other hand, the affinity of compound **5f** was the least with binding energy of −4.15kca/mol with three hydrogen bonds’ formation. In addition to the hydrogen bonding interactions, the docking analysis revealed the presence of other interactions such as hydrophobic interactions and pi–pi and pi-alkyl interactions. In telomeric quadruplex docking also the compound **5d** exhibited most hydrophobic interactions (six) with the DNA base pairs, followed by four interactions seen in compound **5f**. All the compounds exhibited similar or higher number of hydrogen interactions compared to standard **4** with compounds **5b** and **5c** displaying three hydrophobic interactions. Other noncovalent interactions (pi-pi and pi-alkyl) were also observed and most of the compounds exhibited similar or higher number of pi-pi and pi-alkyl interactions compared to standard **4 (**see Supplementary Material).

**TABLE 2:** Molecular docking analysis of some critical parameters of compounds **5a–f** and BMSG-SH-3 (**4**) with telomeric G-quadruplex^a^.

S. No	Compound	Binding affinity (Kcal/mol)	Inhibition constant	Hydrogen bonds	Hydrogen bonds lengths (Angstrom)	No. of hydrophobic interactions^b^	No. of pi-pi and pi-alkyl interactions^c^
1	Control **4**	−3.70	1.95 mM	A:R8G23:CBA - A:DG14:O4′	2.78	1	2
				A:R8G23:CAP - A:DG8:O4′	3.73		
				A:R8G23:CAB - A:DG8:O3′	2.92		
2	**5a**	−5.33	123.82 uM	:UNK1:C37 - A:DG22:O6	2.93	1	3
3	**5b**	−7.82	1.86 uM	A:DG8:H22 - :UNK1:O33	2.37	3	1
				A:DG9:H22 - :UNK1:O1	1.80		
				A:DG9:H3 - :UNK1:O1	2.88		
				A:DG14:C5' - :UNK1:O22	2.88		
4	**5c**	−6.42	19.75 uM	A:DG4:H3 - :UNK1:O58	1.84	3	2
				A:DG4:C1' - :UNK1:O58	2.83		
				:UNK1:C20 - A:DG22:O6	3.74		
5	**5d**	−7.47	3.34 uM	A:DG2:H22 - :UNK1:O48	2.22	6	2
				A:DG2:H3 - :UNK1:O48	2.79		
				A:DG14:C8 - :UNK1:O14	3.67		
				:UNK1:C21 - A:DG14:O4′	2.93		
6	**5e**	−4.81	296.88 uM	:UNK1:C22 - A:DT11:OP1	2.84	1	4
7	**5f**	−4.15	913.04 uM	A:DG10:H22 - :UNK1:O14	2.65	4	2
				A:DG10:H3 - :UNK1:O27	3.01		
				:UNK1:C16 - A:DG16:OP2	2.46		

^a^ See Supplementary Material for more information.

^b,c^ Other non-covalent interactions between ligands and telomeric G-quadruplex.

### Chemical Synthesis

**SCHEME 1:**
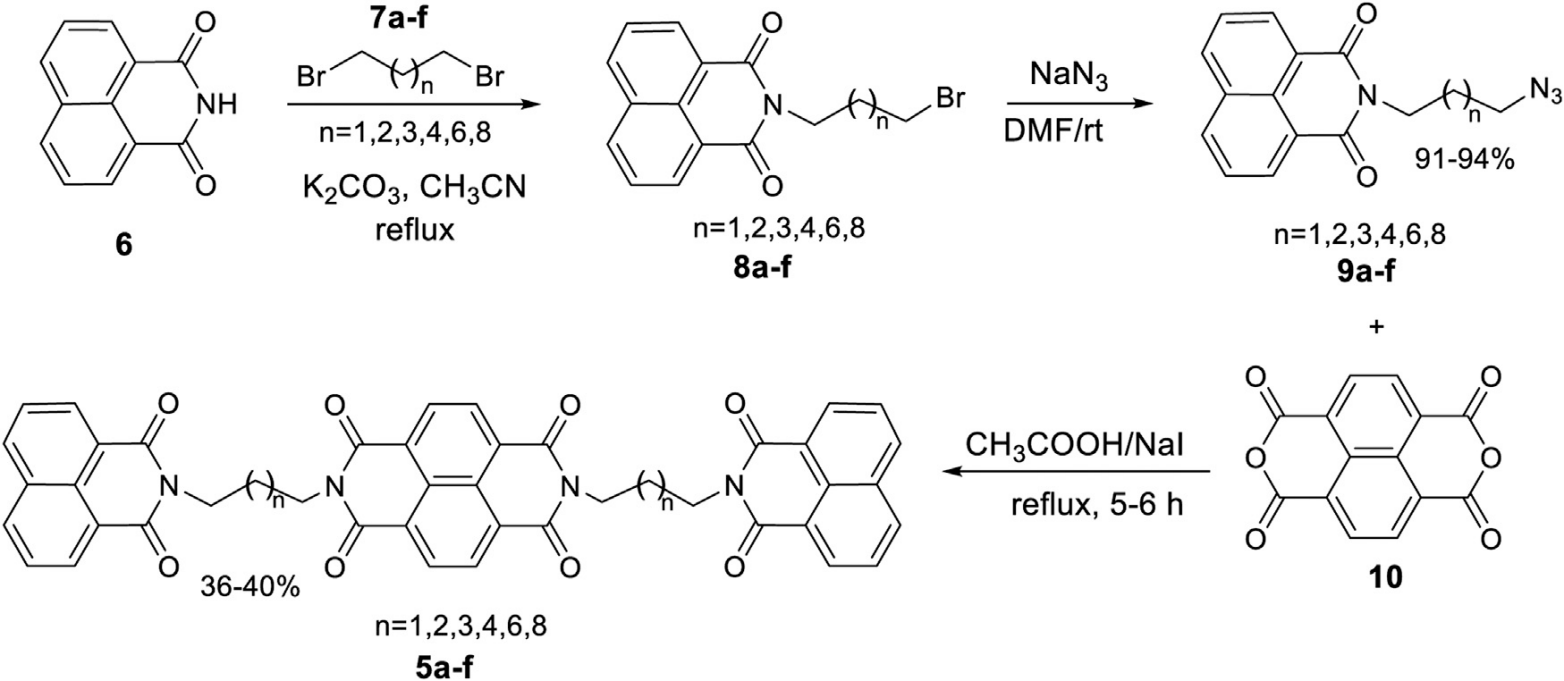
Synthetic route to the desired DNA interactive agents **5a–f**.

Molecular docking analysis of the designed naphthalene diimide linked bis-naphthalimides on DNA intercalation and telomeric DNA quadruplex sites provided excellent results compared to the standard. Therefore, we proceeded to synthesize the compounds and study whether the promising docking results could be reflected on the actual DNA denaturation and cytotoxic properties of the compounds. For this purpose, we designed a convenient synthetic strategy to access the desired molecules **5a–f** (Scheme 1). 1,8-Naphthalimide **6** was used as the starting material and was subjected to alkylation employing dibromoalkanes **7a–f** with varying carbon chain length. The reaction was carried out in the presence of a base with acetonitrile as solvent under refluxing condition to afford *N*-alkyl naphthalimides **8a–f**. The azidation of these *N*-alkyl naphthalimides **8a–f** was done by employing sodium azide in dimethyl formamide as solvent to yield the key intermediates *N*-alkyl azido naphthalimides **9a–f**. The key intermediates **9a–f** were then treated with 1,4,5,8 naphthalic dianhydride in the presence of excess sodium iodide with acetic acid as solvent under reflux condition, to yield the desired naphthalene diimide linked bis-naphthalimides **5a–f**.

### Biological Evaluation

The synthesized naphthalene diimide linked bis-naphthalimides **5a–f** were evaluated for their *in vitro* DNA binding ability to substantiate the molecular docking results, followed by assessment of cytotoxic properties against a panel of human cancer cell lines.

#### Thermal Denaturation Studies

Chemical entities that bind preferentially to DNA duplex are known to elevate the melting temperature (*T*m) of DNA by stabilizing the double helix. This thermal denaturation analysis is a straightforward way to demonstrate the binding of drug to the DNA. The DNA binding ability for the compounds **5a–f** was determined by conducting thermal denaturation studies using duplex-form calf thymus (CT) DNA (Kamal et al., 2002). These studies were carried out at 1:5 molar ratio of compounds **5a–f**/DNA and all the compounds elevated the helix melting temperature of CT-DNA (Table 3). These compounds showed a change in melting temperature (∆*T*_m_) in the range of 0.3–1.0°C at 0, 18, and 36 h time intervals (Table 3). No significant change in *T*_m_ was observed after 36 h incubation of compounds with DNA. Among the series, compounds **5b** and **5c** displayed better ∆*T*_m_ than the naturally occurring DC-81, which is usually used as a standard in thermal denaturation studies. The compounds **5b** and **5c** exhibited ∆*T*_m_ of 0.7°C at 18 h of incubation time; however after 36 h of incubation ∆*T*m was better than standard DC-81. The compound **5b** was the best that exhibited ∆*T*_m_ of 1.0°C and a change in thermal denaturation of 0.8°C was observed with compound **5c**. It is interesting to note that among all the compounds, **5b** and **5c** exhibited best binding affinities toward the DNA helices in the docking studies. Moreover, the results validate the promising DNA interactive properties of the compounds **5b** and **5c.**

**TABLE 3:** Thermal denaturation studies of compounds **5a–f** using duplex-form calf thymus (CT) DNA at 1:5 molar ratio.

Compound	Compound:[DNA] molar ratio^b^	*∆T*m (°C)^a^ after incubation at 37°C		
		0 h	18 h	36 h
**5a**	1:5	0.3	0.3	0.4
**5b**	1:5	0.5	0.7	1.0
**5c**	1:5	0.6	0.7	0.8
**5d**	1:5	0.3	0.4	0.6
**5e**	1:5	0.4	0.5	0.5
**5f**	1:5	0.3	0.3	0.4
DC-81	1:5	0.3	0.7	0.7

^a^ For CT-DNA alone at pH 7.00 ± 0.01, *T*m = 69.2°C ± 0.01 (mean value from 10 separate determinations), all ∆*T*m values are ±0.01–0.02°C.

^b^ For a 1:5 molar ratio of [compound **5a–f**]/[DNA], where CT-DNA concentration = 100 μM and ligand concentration = 20 μM in aqueous sodium phosphate buffer [10 mM sodium phosphate +1 mM EDTA, pH 7.00 ± 0.01].

### Cytotoxicity

The computational and DNA thermal denaturation studies revealed broadly that naphthalene diimide linked bis-naphthalimides with the short chain spacers exhibit better DNA interactive ability. We then proceeded to evaluate whether the DNA interactive ability of the **5a–f** could induce cytotoxic effects against human cancer cell line. The compound **5b** was selected and evaluated for anticancer screening at National Cancer Institute (NCI), United States. It was screened against a panel of 60 human tumor cell lines organized in subpanels representing melanoma, leukemia, and cancers of breast, prostate, lung, colon, ovary, CNS, and kidney by using sulforhodamine B (SRB) method at single dose concentration of 10^−5^ M. Compounds which reduced the growth of the cell lines to 50% or less compared with untreated control cells at this concentration were considered as active against the corresponding cell lines. The compound **5b** exhibited significant activity selectively against non-small cell lung cancer and leukemia cell lines as shown in Table 4. A percent growth of 45.42 was seen in HOP-92 lung cancer cell line and in case of leukemia, percent growth of 23.93 and 44.91 was observed in cell lines MOLT-4 and SR, respectively.

**TABLE 4:** Growth percent of compounds **5b** in selected cell lines in National Cancer Institute single dose screening^a^ (10^−5^ M).

Cell lines		5b (NSC 745356)
NSCLC^b^	HOP-92	45.42
Leukemia	MOLT-4	23.93
	SR	44.91

^a^ Growth relative to the no-drug control.

^b^ Non-small cell lung cancer cell line.

In addition to this, all the compounds **5a–f** were tested against ten human tumor cell lines organized in subpanels representing cancers of breast, prostate, lung, colon, ovary, and neuroblastoma at two different concentrations, i.e.,10^−5^ M and 10^−6^ M, and the results are shown in Table 5. All the tested compounds exhibited little activity against cell lines of prostrate, neuroblastoma, liver, and ovary cancer. Some activity was observed in MCF-7 breast cancer cell line and HCT-15 colon cancer cell lines. The compounds **5a–f** exhibited some promising activity against colon-205 and A549 lung cancer cell lines. Among the series, compounds **5a** and **5b** with short chain spacers displayed good growth inhibition of around 45% against A459 lung cancer at 10 μM concentration. On the other hand, compounds **5e** and **5f** showed 40% and 41% growth inhibitions against A549 and colo-205, respectively.

**TABLE 5:** Cytotoxicity assay of compounds **5a–f** on selected cancer cell lines employing sulforhodamine B (SRB) assay at 10^−5^ M and 10^−6^ M concentrations.

% Growth inhibition^a^											
Cell line type		Prostrate		Colon			Breast	NB^b^	Liver	Ovary	Lung
Compound	μM	DU 145	PC-3	Colo-205	HCT-15	502713	MCF-7	IMR-32	Hep-2	OVCAR-5	A549
**5a**	1	<5	<5	16	17	<5	<10	<5	<5	<5	10
	10	<10	40	35	18	<10	18	<10	<5	<5	42
**5b**	1	<5	<5	21	14	<5	<10	<5	<5	<5	15
	10	<5	<5	34	16	<5	20	<5	<5	<10	45
**5c**	1	<5	<5	22	14	<5	18	<5	<5	<5	19
	10	<10	18	23	16	<5	23	<10	<5	12	30
**5d**	1	<5	<5	8	27	<5	10	15	<5	<5	<10
	10	<10	<10	19	28	<5	15	25	17	18	20
**5e**	1	<5	<5	26	19	<5	25	<10	<5	<5	21
	10	<5	22	36	31	<5	29	13	46	15	40
**5f**	1	10	<5	38	25	<5	25	15	<5	<5	26
	10	18	34	41	27	<5	29	17	30	14	35
5FU	2	47	53	65	58	80	98	48	39	35	52

^a^ Assay was carried out in triplicate and the growth inhibition exhibited SD values in the range of ±0.4–1.2.

^b^ Neuroblastoma.

### ADME Computational Analysis

The pharmacokinetic and pharmacodynamic properties of the compounds reveal whether a chemical compound has potential to be developed as a clinical drug. *In silico* ADME predictions provide an insight into these critical properties prior to conducting the actual *in vitro* and *in vivo* studies. The druglikeness and ADME properties of compounds **5a**–**f** were predicted by using preADMET and SwissADME online tools and compared with standard **3**, which reached clinical studies (Table 6). The predicted Caco2 values revealed that all the compounds along with the standard exhibited similar biological barrier crossing ability and were not blood brain barrier permeant. However, the compounds acted as inhibitor of P-gp, a transporter protein, that could result in decreased bioavailability. This was further validated by high TPSA and low bioavailability scores. iLOGP is a critical physiochemical parameter in drug design and is one of the factors contributing to the drug availability. Interestingly, the compounds **5a** and **5b** exhibited iLOGP values comparable to the standard 3. Both the compounds are promising candidate to be taken further for development by structural diversification.

**TABLE 6:** Some selected ADME predictions of compounds **5a**–**f** using preADMET and SwissADME.

Compound	**5a**	**5b**	**5c**	**5d**	**5e**	**5f**	**3**
BBB permeant	No	No	No	No	No	No	No
Caco2	21.1625	20.8232	21.2759	21.1915	21.3918	21.6771	21.1732
Pgp_inhibition	Inhibitor	Inhibitor	Inhibitor	Inhibitor	Inhibitor	Inhibitor	Non
Skin_Permeability	−3.66536	−3.19396	−2.7807	−2.42649	−2.15702	−1.83794	−3.82532
#Rotatable bonds	8	10	12	14	16	20	10
#H-bond acceptors/donors	8/0	8/0	8/0	8/0	8/0	8/0	6/2
TPSA	156.28	156.28	156.28	156.28	156.28	156.28	102.2
iLOGP	4.53	5.09	5.59	6.11	6.62	7.5	4.24
Log Kp (cm/s)	−6.78	−6.45	−6.11	−5.77	−5.18	−3.98	−6.94
Bioavailability score	0.17	0.17	0.17	0.17	0.17	0.17	0.55

## Conclusion

Bis-naphthalimide and naphthalene diimide motifs are important pharmacophores in anticancer drug design. A molecular modeling assisted rational design of new naphthalene diimide linked bis-naphthalimides as potential DNA interactive anticancer agents was undertaken. These compounds were subjected to molecular docking for their DNA intercalating and telomeric G-quadruplex binding potential. Compounds **5a** and **5b** provided excellent results for DNA intercalation and G-quadruplex binding, respectively, compared to the standards. This encouraged us to synthesize these compounds and study their *in vitro* DNA interactive ability and cytotoxicity. The compound **5b** stabilized the CT-DNA better than the standard and exhibited promising anticancer activity against HOP-92, A549 (lung cancer), MOLT-4, and SR (leukemia). Similarly, **5a** also exhibited decent activity against few cancer cell lines. A prediction of ADME properties was undertaken to understand the exhibited cytotoxicity of the compounds and thereby identify a potential molecule for further development. Based on all the studies, it was found that naphthalene diimide linked bis-naphthalimides with short chain spacers have potential for further development. Notwithstanding the excellent computational and promising experimental results suggesting DNA binding ability, the lower cytotoxicity of these compounds against cancer cell lines could be attributed to decreased bioavailability as shown in ADME predictions. However, a more focused chemical diversification of compounds **5a** and **5b** to overcome the bioavailability issues could lead to more potent molecules.

## Data Availability

The original contributions presented in the study are included in the article/Supplementary Material; further inquiries can be directed to the corresponding authors.
