# Supplementary material for: Rational Design and Synthesis of Naphthalene Diimide Linked Bis-Naphthalimides as DNA Interactive Agents
Source: Front Chem. 2021 Mar 10;9:630357. doi: 10.3389/fchem.2021.630357 (PMC7988716; doi:10.3389/fchem.2021.630357)
Supplement: Supplementary file 1 [file DataSheet1.PDF]

Supporting information for:

Rational Design and Synthesis of Naphthalene diimide linked Bisnaphthalimides as DNA Interactive Agents

M. Shaheer Malik[1*], Syed Farooq Adil[2], Ziad Moussa[3], Hatem M. Altass[1], Ismail I. Althagafi[1], Moataz Morad[1], Mohammad Azam Ansari[4], Qazi Mohammad Sajid Jamal[5], Rami J. Obaid[1], Abdulrahman A. Al-Warthan[2], Thokhir B. Shaik[6], Saleh A. Ahmed[1,7,8*]

[1]*Department of Chemistry, Faculty of Applied Sciences, Umm Al-Qura University, Makkah 21955, Saudi Arabia*
[2]*Department of Chemistry, College of Science, King Saud University, Riyadh 11451, Saudi Arabia*
[3]*Department of Chemistry, College of Science, United Arab Emirates University, P.O. Box 15551, Al Ain, Abu Dhabi, United Arab Emirates*
[4] *Department of Epidemic Disease Research, Institute for Research & Medical Consultations (IRMC), Imam Abdulrahman Bin Faisal University, Dammam 31441, Saudi Arabia*
[5] *Department of Health Informatics, College of Public Health and Health Informatics, Qassim University, Al Bukayriyah, Saudi Arabia*
[6]*Research on Advanced BioMedical Solutions Pvt Ltd, KPHB, Hyderabad 500071, India*
[7]*Department of Chemistry, Faculty of Science, Assiut University, 71516 Assiut, Egypt*
[8]*Research laboratories unit, Faculty of Applied Science, Umm Al-Qura University, 21955 Makkah, Saudi Arabia*

Correspondence may be addressed to:

M. Shaheer Malik: E-mail address: msmalik@uqu.edu.sa ; Saleh A. Ahmed: E-mail address: saahmed@uqu.edu.sa

Contents:

- Non-covalent interactions between compounds **5a-f** and d(atgcat)2 site of DNA
- 2D Docking structures of selected compounds at d(atgcat)2 site of DNA
- Non-covalent interactions between compound **5a-f** and telomeric G-quadruplex
- 2D Docking structures of selected compounds with telomeric G-quadruplex
- One dose graph of percent growth of compound **5b** against NCI-60 Human Tumor Cell Lines Screen
- Spectra of selected compounds

Molecular modelling analysis revealing DNA intercalation

S.No.	Compounds	Residues involved in hydrophobic interaction	Residues involved in Pi-Pi interaction/Pi-Alkyl
1	Control **3**	B:DG3, A:DA5, A:DG3	A:DC4, A:DT2,B:DA5,B:DC4
2	**5a**	B:DC4 and B:DA5	A:DT2,A:DA5,B:DB3, A:DC:3
3	**5b**	--	B:DT2,B:DG3, A:DC4,
4	**5c**	A:DG3,A:DT6 and B:DA1	A:DC4,B:DG3, B:DT2
5	**5d**	A:DT6, A:DC4, B:DC4 and B:DA5	B:DG3,B:DT2
6	**5e**	A:DT6 and B:DA1	A:DT2, A:DA5, B:DT2, B:DG3
7	**5f**	A:DT2, B:DC4	A:DA5,B:DG3, B:DA5

where A and B are the DNA helix chains

Table: Non-covalent interactions between compounds **5a-f** and d(atgcat)2 site of DNA

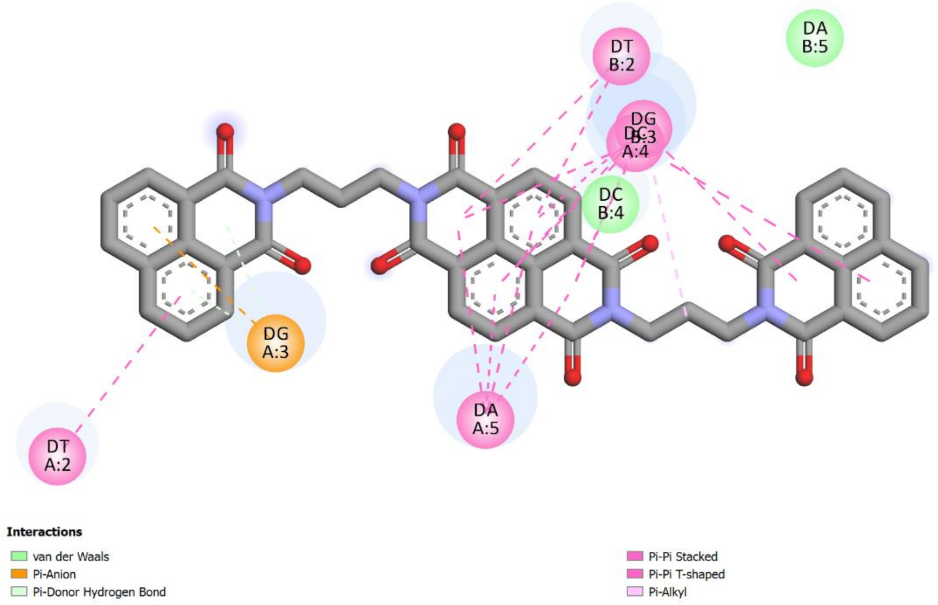

2D Docking structure of compound **5a** at d(atgcat)2 site of DNA

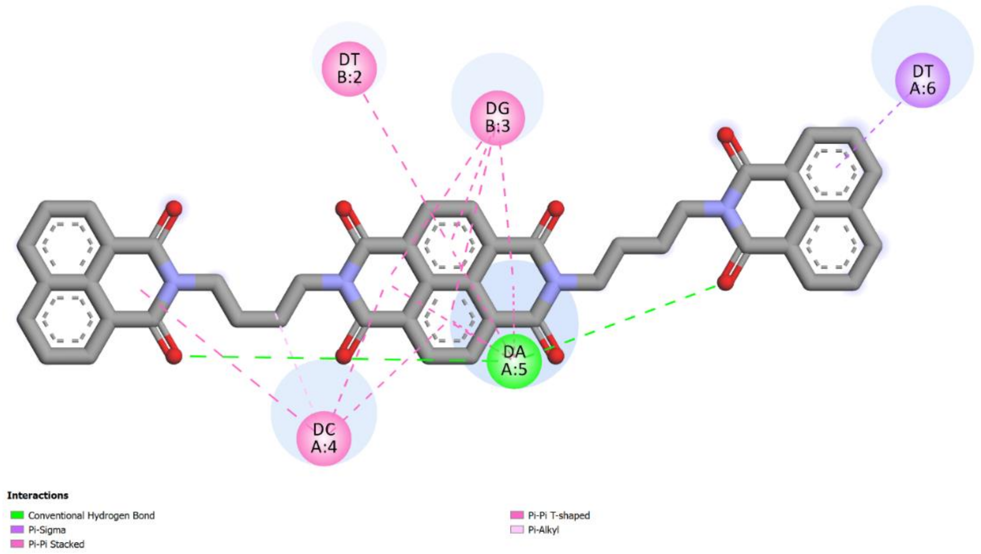

2D Docking structure of compound **5b** at d(atgcat)2 site of DNA

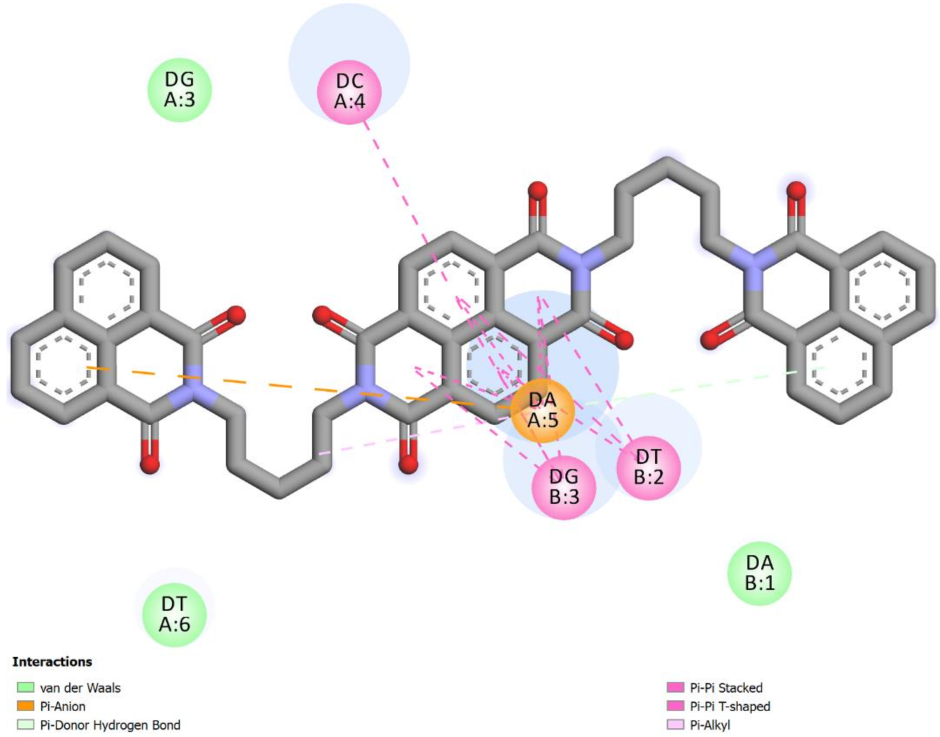

2D Docking structure of compound **5c** at d(atgcat)2 site of DNA

Molecular modelling analysis revealing interaction with telomeric G-quadruplex

S.No.	Compounds	Residues involved in hydrophobic interaction	Residues involved in Pi-Pi interaction/Pi-Alkyl
1	Control **4**	A:DG9	A:DG2, A:DG20
2	**5a**	A:DG22	A:DG16, A:DG4, A:DG:10
3	**5b**	A:DG14, A:DG10, A:DT12	A:DT11
4	**5c**	A:DG8, A:DG3, A:DG22	A: DG10, A:DG16
5	**5d**	A:DG2, A:DG9, A:DG10, A:DT12, A:DT11, A:DG14	A:DG20, A:DG8
6	**5e**	A:DT11	A:DG4,A:DG10, A:DG16, A:DG22
7	**5f**	A:DT11, A:DT16, A:DT17, A:DG15	A:DG4, A:DG22

Where A is DNA helix Chain

Table: Non-covalent interactions between compound **5a-f** and telomeric G-quadruplex

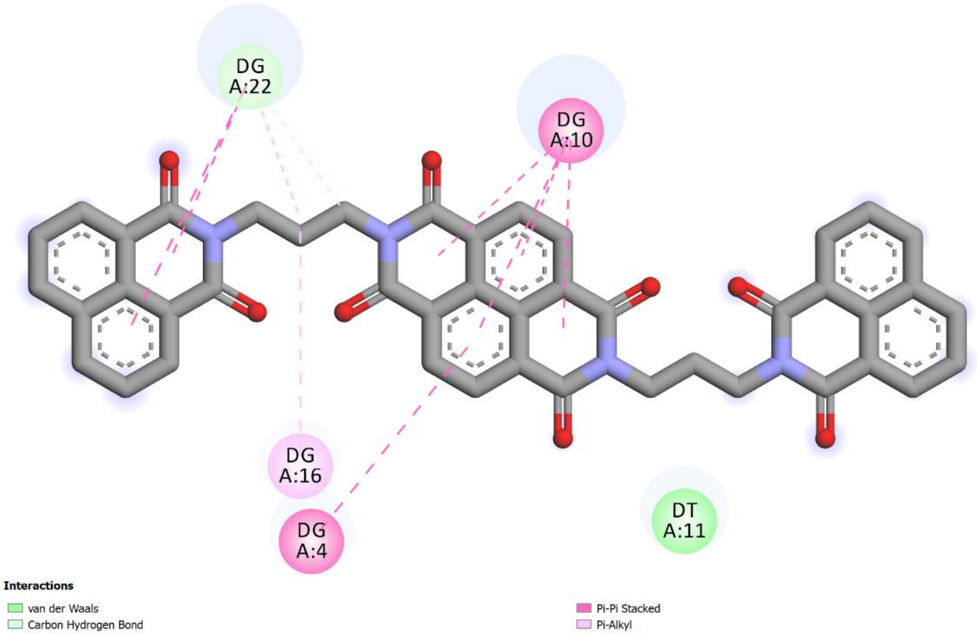

2D Docking structure of compound **5a** with telomeric G-quadruplex

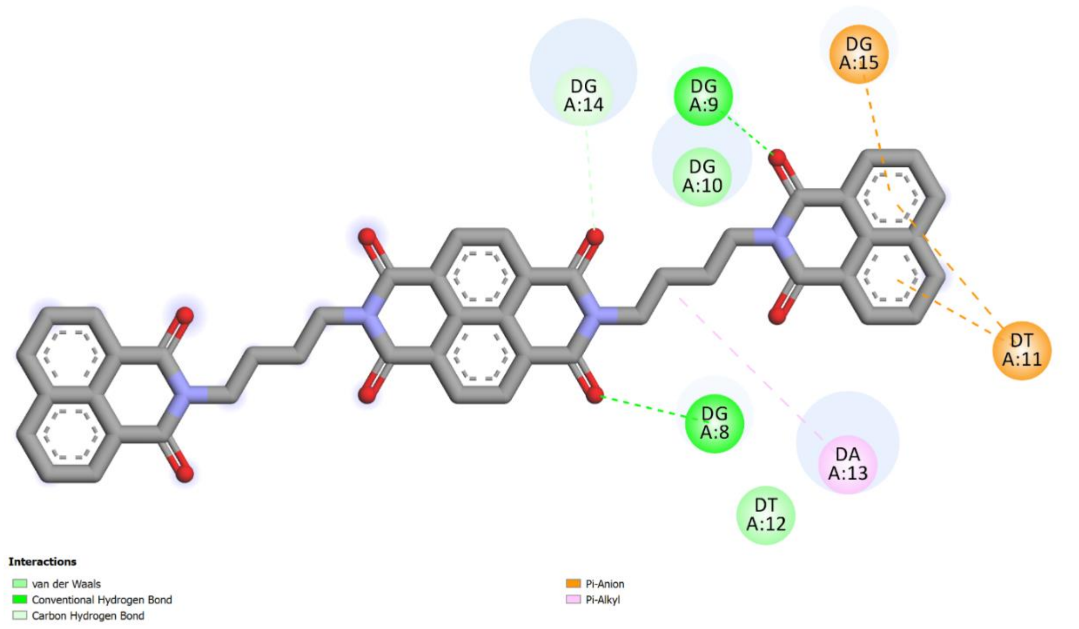

2D Docking structure of compound **5b** with telomeric G-quadruplex

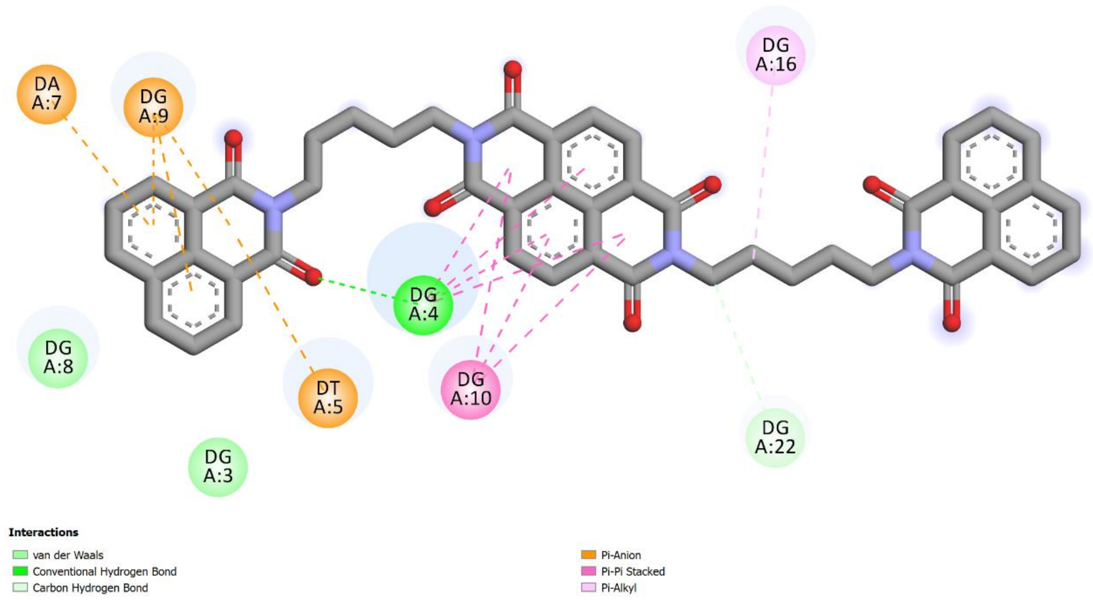

2D Docking structure of compound **5c** with telomeric G-quadruplex

One Dose Data Graph for NSC 745356

DTP OneDose/Syn/60 Cell Line

Cell Panel	Cell Line	Growth Percent
Leukemia	HL-60(TB)	89.4
	K-562	93.9
	MOLT-4	23.9
	RPMI-8226	121.5
	SR	44.9
Non-Small Cell Lung	A549/ATCC	100.0
	EKVX	103.2
	HOP-62	114.4
	HOP-92	45.4
	NCI-H226	106.1
	NCI-H23	129.7
	NCI-H322M	223.8
	NCI-H460	105.3
	NCI-H522	113.9
Colon	COLO 205	136.8
	HCC-2998	126.6
	HCT-116	111.7
	HCT-15	114.6
	HT29	118.1
	KM12	108.6
	SW-620	118.1
Central Nervous System	SF-268	101.7
	SF-295	146.7
	SF-539	100.1
	SNB-19	108.7
	SNB-75	109.0
	U251	115.2
Melanoma	LOX IMVI	118.6
	MALME-3M	298.1
	M14	126.9
	MDA-MB-435	122.0
	SK-MEL-2	104.4
	SK-MEL-28	127.0
	SK-MEL-5	95.9
	UACC-257	126.1
	UACC-62	101.5
Ovarian	IGROV1	106.7
	OVCAR-3	118.6
	OVCAR-4	125.9
	OVCAR-5	123.7
	OVCAR-8	96.9
	NCI/ADR-RES	110.9
	SK-OV-3	122.0
Renal	786-0	96.4
	A498	104.6
	ACHN	120.7
	CAKI-1	136.5
	SN12C	85.9
	TK-10	198.6
	UO-31	98.2
Prostate	PC-3	106.7
	DU-145	104.9
Breast	MCF7	92.0
	MDA-MB-231/ATCC	117.2
	HS 578T	116.4
	BT-549	224.3
	T-47D	115.6
	MDA-MB-468	112.5

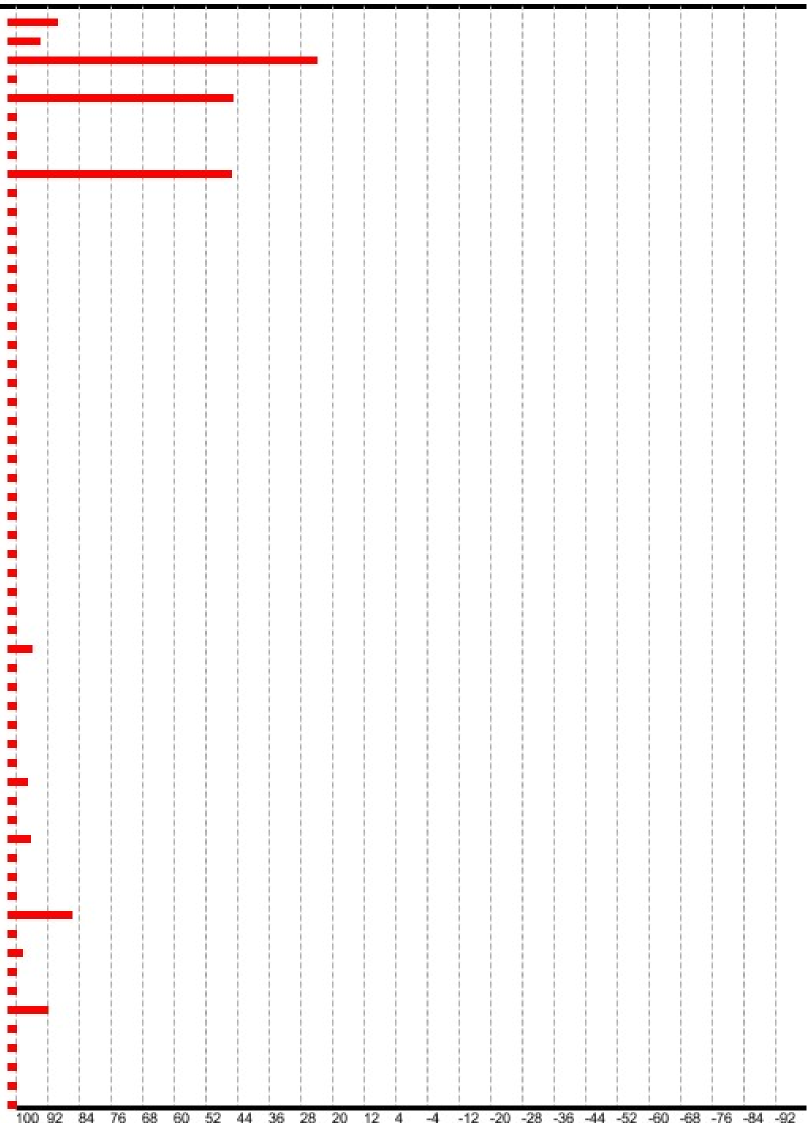

Growth percent of compound **5b** against NCI-60 Human Tumor Cell Lines Screen conducted by Development Therapeutics Program, National Cancer Institute, USA.

Spectroscopic characterization

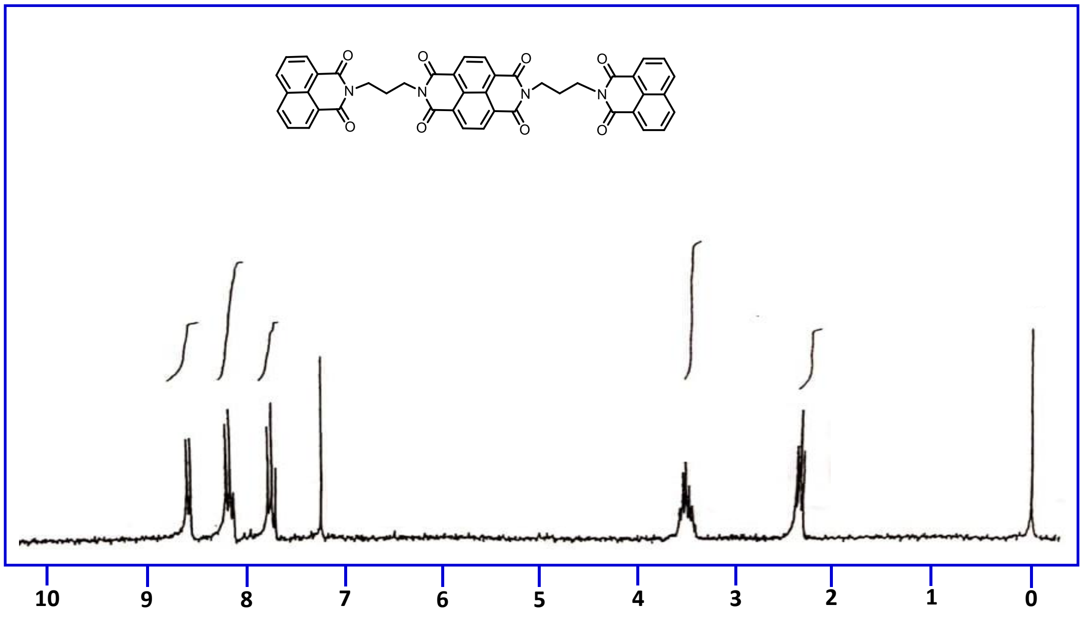

^{1}H NMR spectrum of compound **5a**

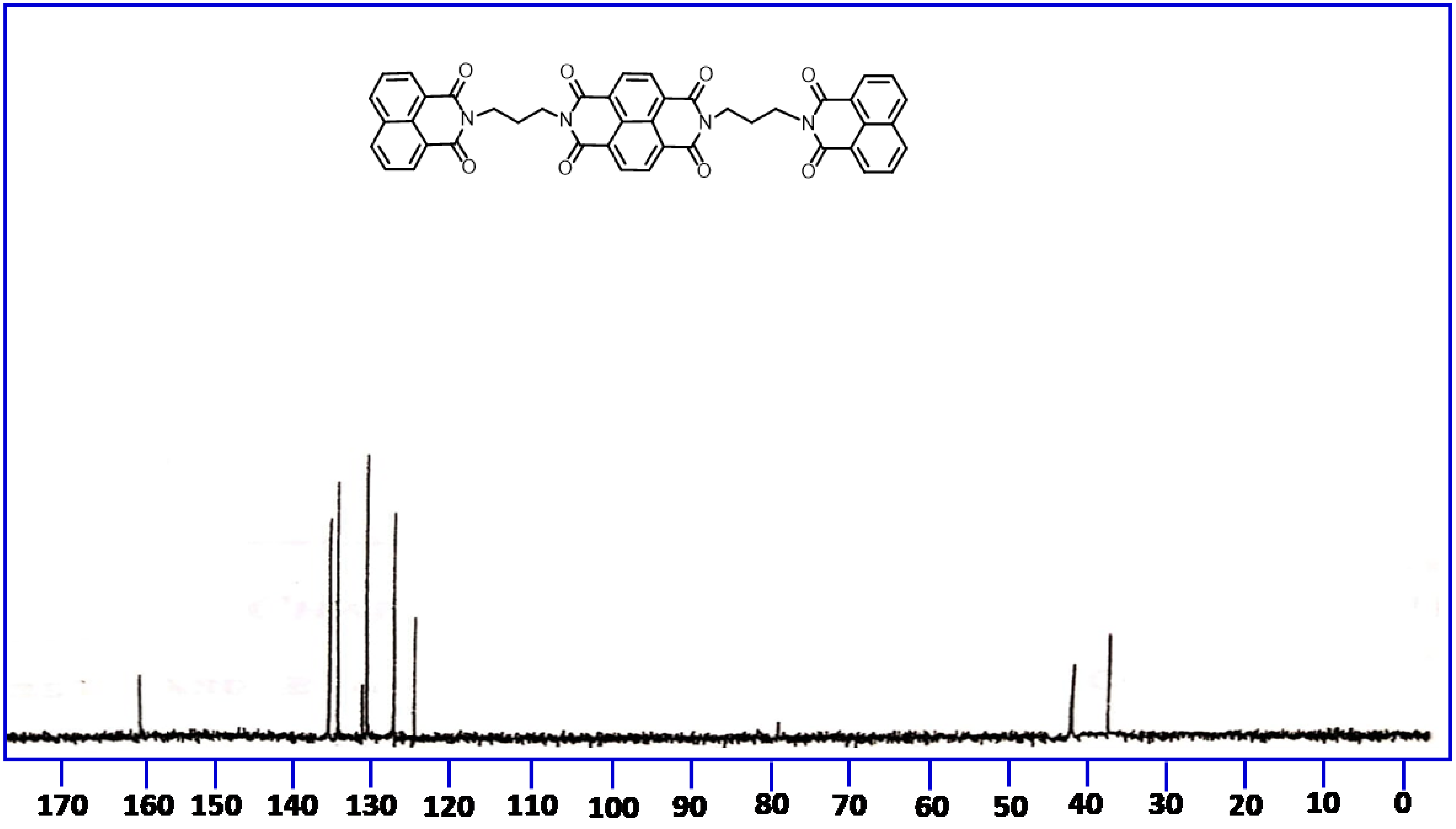

^{13}C NMR spectrum of compound **5a**

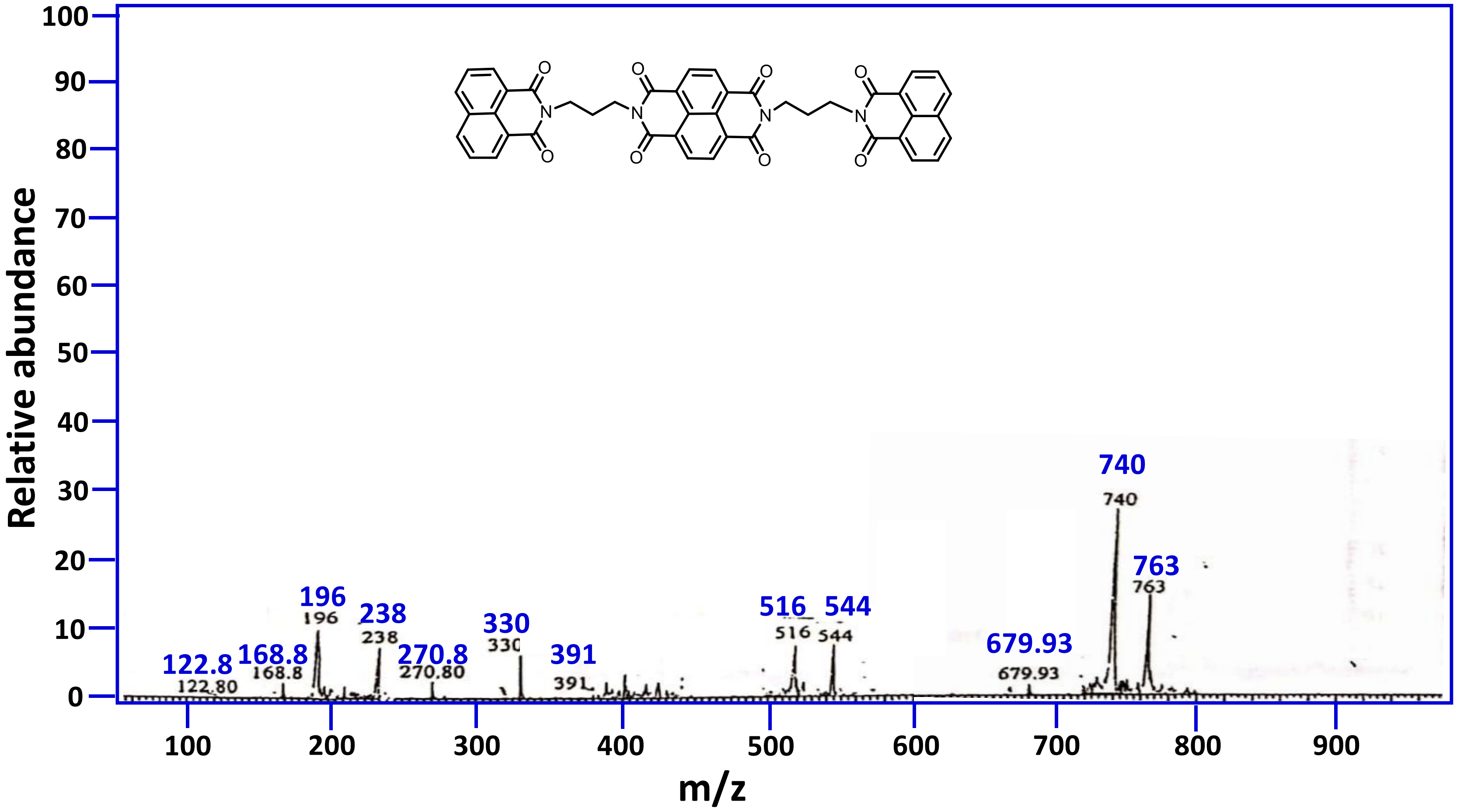

Mass spectrum of compound **5a**

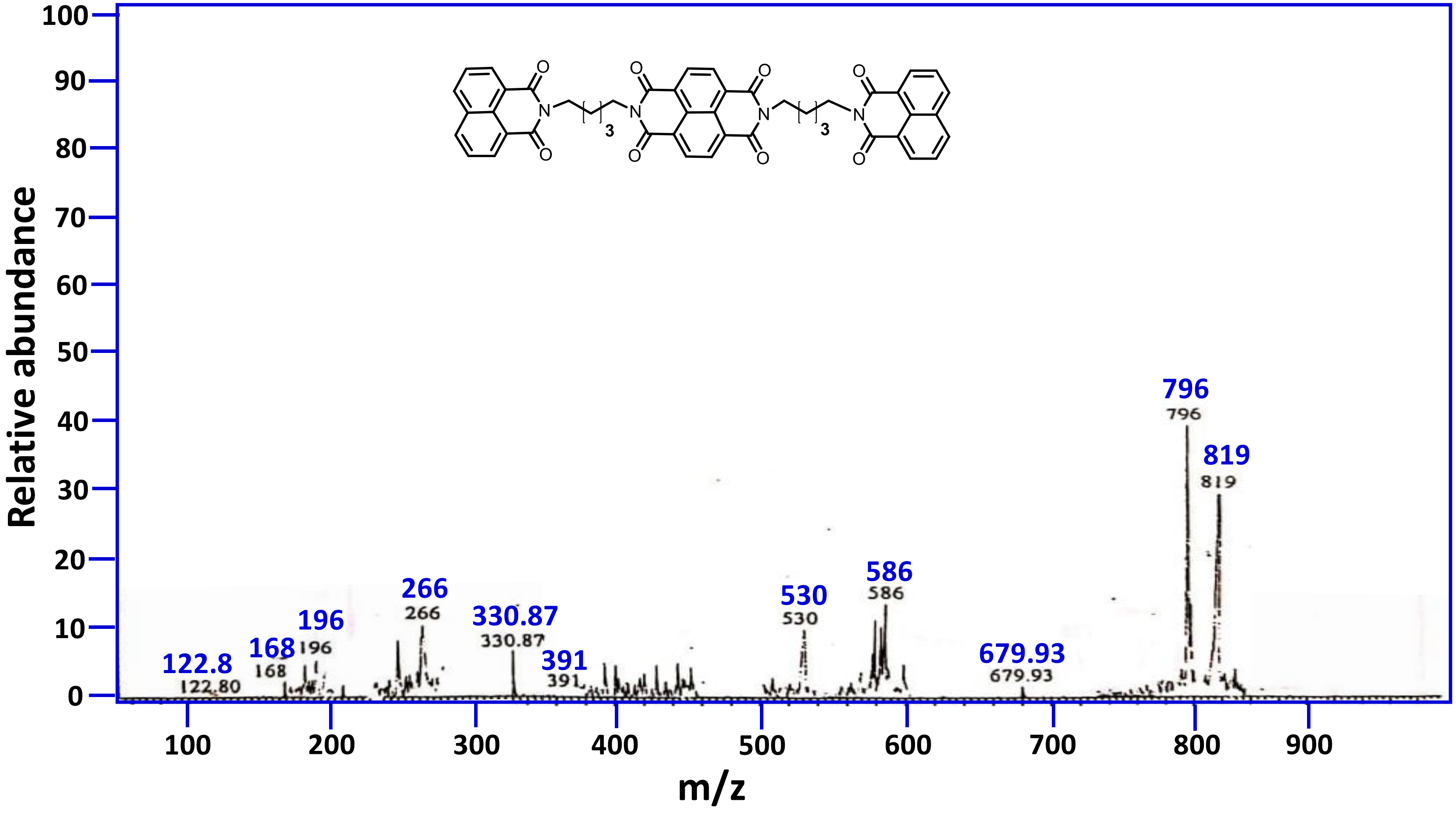

Mass spectrum of compound **5c**

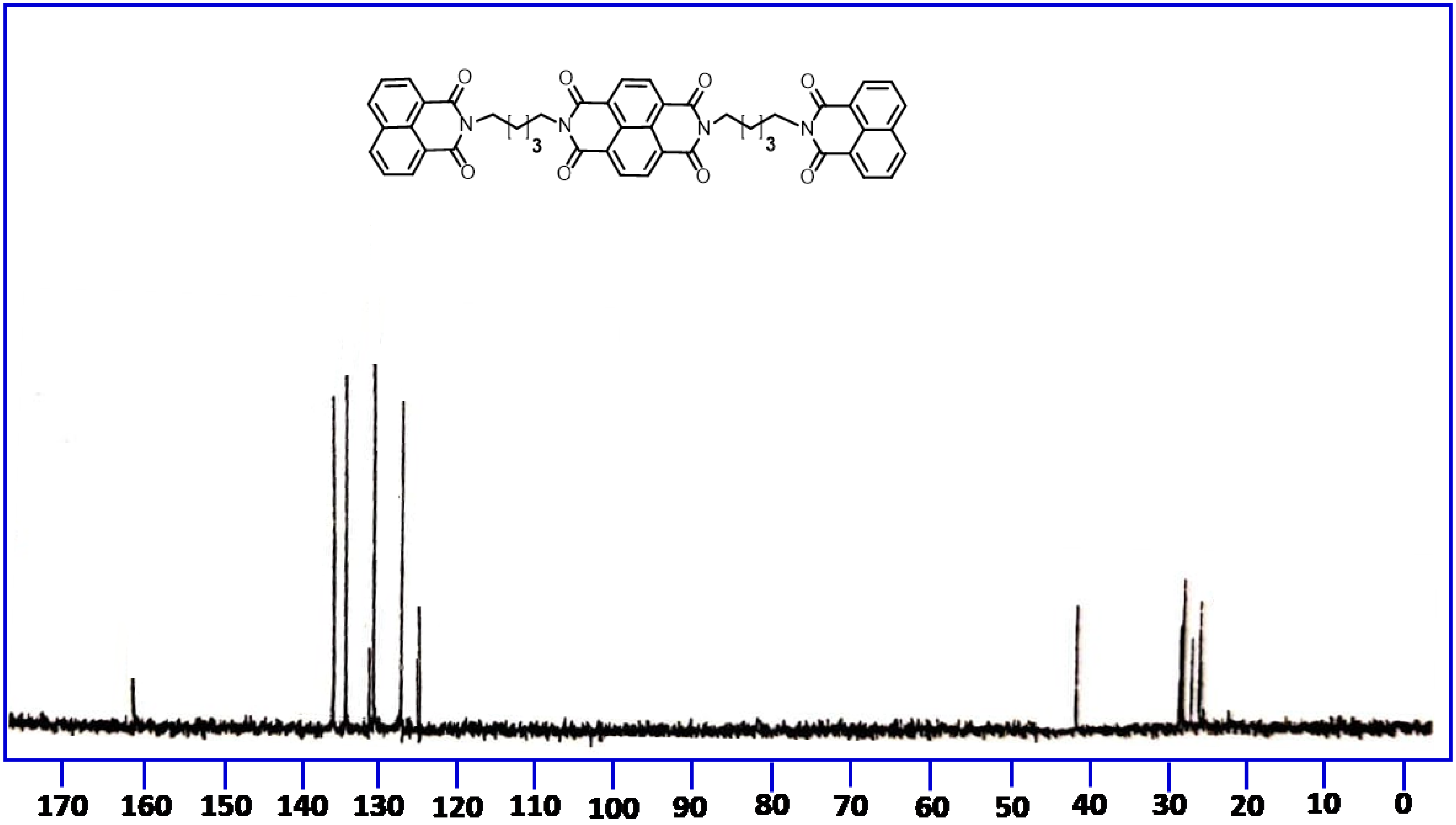

^{13}C NMR spectrum of compound **5c**

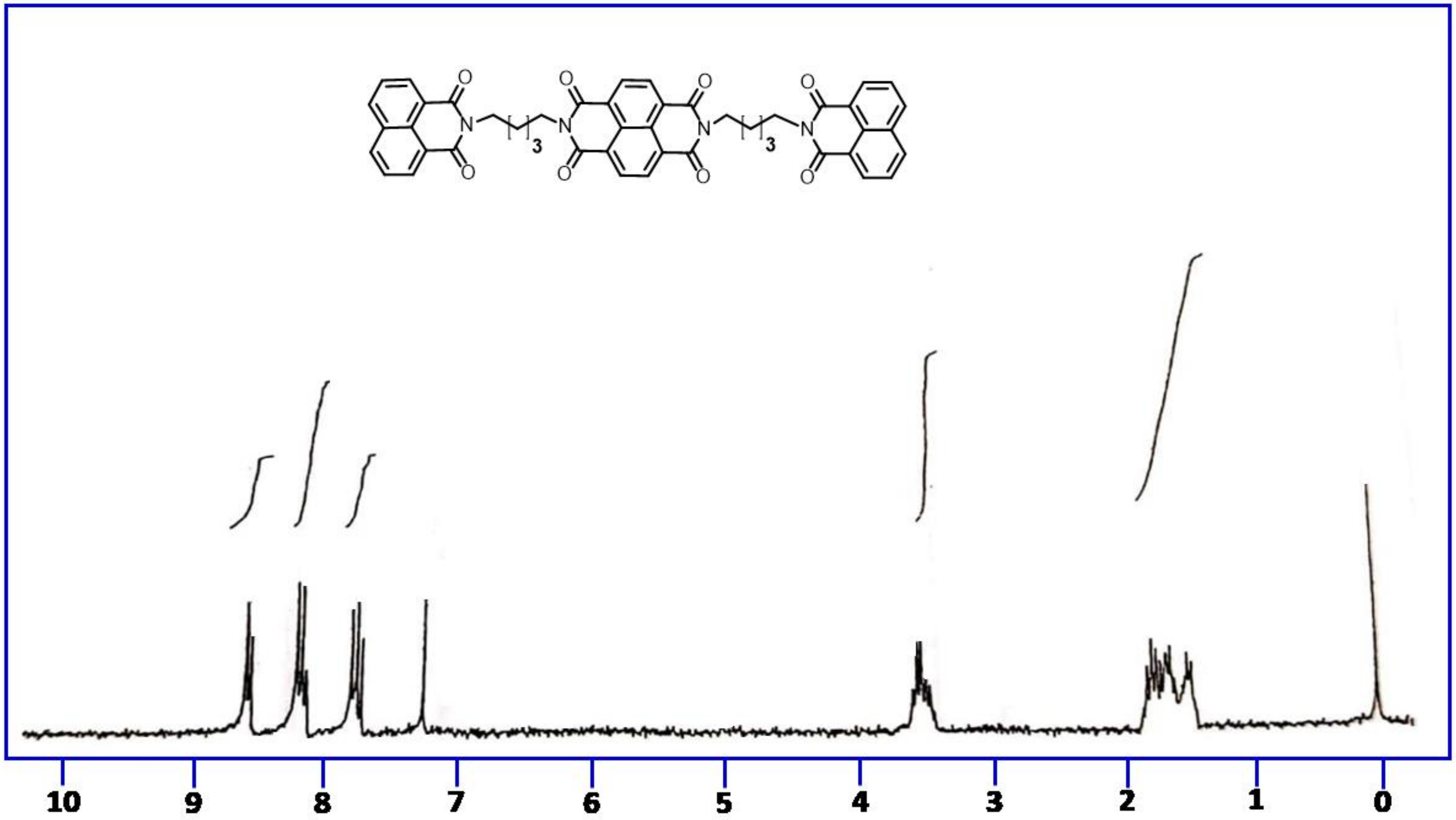

^{1}H NMR spectrum of compound **5c**
