# Supplementary material for: Rational Design and Synthesis of Naphthalene Diimide Linked Bis-Naphthalimides as DNA Interactive Agents
Source: Front Chem. 2021 Mar 10;9:630357. doi: 10.3389/fchem.2021.630357 (PMC7988716; doi:10.3389/fchem.2021.630357)
Supplement: Supplementary file 2 [file Table1.DOCX]

**Prof. Dr. Saleh A. Ahmed	Phone: +966 (0) 530435760**

***Permanent address:* Email:** [**saahmed@uqu,edu.sa**](mailto:saahmed@uqu,edu.sa)

***Chemistry Department*	saleh.a.ahmed@aun.edu.eg,**

***Faculty of Science*	saleh_63@hotmail.com**

***Assiut University***

***71516 Assiut***

***EGYPT***

**To**

**Prof. Dr. Steve Suib**

Editor-in-Chief, Frontiers in Chemistry

University of Connecticut
Mansfield, United States

## **------------------------------------------------------------------------------------------ *Dear respected Prof. Dr.*** [***Suib***](https://loop.frontiersin.org/people/60186/overview)

First of all, I would like to thank you so much for your kind co-operation. As invited by **Professor** Na'il Saleh to contribute in your respected journal, I am very pleased to send you the revised manuscript entitled:

“Rational Design and Synthesis of Naphthalene diimide linked Bisnaphthalimides as DNA Interactive Agents”

We are highly indebted to your kindness and non-stopping help and support. We are pleased to send you the revised manuscript after taking into account all the referees’ comments. **As you may kindly recognize, the reviewers have appreciated the work and recommended publication after minor revision and addressing some issues**. We have addressed all other queries raised by the learned reviewers. The constructive comments from the reviewers have facilitated further improvement of the quality of the manuscript. In the following section, we have addressed the queries of the reviewer point by point (answers in red-italic font) and **yellow highlighting** in the manuscript text. Hope this will be satisfactory to warrant publication.

Best regards and all the best

 Sincerely yours,

**Prof. Saleh A. Ahmed**

**Response to Reviewers**

**Reviewer 1:**

Though the study is carried out well, there are few quarries that need to be explained

We thank the reviewer for his valuable comments which have taken seriously, constructively and addressed as below:

1. In line -233, the pH should be mentioned as ± 0.01.

The required correction is incorporated in the manuscript.

2.In table. 3," compound" should be mentioned, not as "Compd".
The suggested correction is incorporated in the manuscript.

3. Under materials and methods, the cell lines names that are used in the study, should be mentioned under the respective table.

As suggested by the esteemed reviewer, the cell lines used in the study are mentioned in the material and methods section and under the respective table.

4. For each table and figure, the legends are provided very briefly. They should be given in detailed. Legends should be self-explanatory. It should reflect the informations like, the main purpose of doing that assay, the conditions maintained and concentration of compound/DNA taken for the assay should be mentioned.

As suggested by the esteemed reviewer, the legends are rewritten to be more informative.

5.The authors should check the heading for the table 4 is mentioned as "Percent growth of compounds 5b in cell lines" but it should be "Percentage of cell lines growth in presence of compound 5b"

The heading of the table 4 is rewritten as “Growth percent of compounds **5b** in selected cell lines in National Cancer Institute single dose screening^”^. The “Growth percent” term is used by the National cancer institute and the growth percent graph is incorporated in the supplementary file.

6.The table 5 should be corrected. Usually, the cytotoxicity assay will be carried out 3-4 times and the average values will be provided. But I do not know how many times the assay was done in the present study. The values should be represented as "±" some value as it is a mean average of some value

The assay was performed in triplicate and it is appropriately mentioned in the material and method section. Moreover, as suggested by the esteemed reviewer, we have represented the SD values as “±”.

7.Under the table 5 the details of each cell line should be given.

The details of the abbreviation of cell line are provided under the table.

8. It is not clear why telomeric G-quadruplex DNA was considered for modelling studies, whereas for thermal denaturation studies, double stranded DNA namely CT- DNA was considered. Why for thermal denaturation experiments also telomeric G-quadruplex DNA is not considered.

We thank the reviewer for this insightful comment. The initial intent was to develop the chemical templates as DNA intercalators and the initial results encouraged us to undertook the molecular modeling studies with telomeric G-quadruplex DNA as the designed chemical templates has naphthalene diimides (NDIs) as one of the core constituents. The NDIs are well established telomeric G-quadrulex binders and this is also discussed in the manuscript. We agree with the reviewer that a thermal denaturation experiments with telomeric G-quadruplex DNA is not being considered at this point. This is owing to limited resources in this unprecedented situation. The conducted study led to identification of couple of promising compounds for further development and this helpful suggestion of the reviewer with be taken forward in the near future.

9. In the cytotoxicity assay different kinds of cancer cell lines were considered, but it should have one non-cancerous cell line as a positive control.

We thank the reviewer for this comment. The results obtained are promising and which will be taken for further development. However, the compounds are not highly cytotoxic to have any meaningful effect on normal cell line. The observation of the reviewer will be taken forward for investigation at a later stage.

10. In the same manner, since the compounds were dissolved in DMSO, an internal control with only DMSO without any compound should be considered as a control to check the effect of DMSO alone on the cell lines considered in the study.

We agree with the reviewer and this has been appropriately mentioned/clarified in the cytotoxicity assay under the material and method section. The compound stocks were prepared in DMSO where the final DMSO concentration at time of inoculation was maintained below 5% (considered as non-toxic to the cancer cells). We have maintained the DMSO control where the equal amount of DMSO was added in to the control wells. The NCI protocol was followed for this assay where the DMSO was used as the control [Vichai, V. and Kirtikara, K., 2006. Sulforhodamine B colorimetric assay for cytotoxicity screening. *Nature protocols*, *1*(3), pp.1112-1116].

11. Besides the thermal denaturation studies, it will be good to carryout few biophysical experiments to understand the interaction of 5b with both CT DNA and Telomeric G-quadruplex DNA.
We agree with reviewer for few additional biophysical experiments but owing to the prevailing pandemic scenario our labs are not completely functional due to limited access to the wet lab, lack of personnel and vendors. In view of this, we request the reviewer to kindly perceive this as an independent observation and the valuable suggestions will be taken forward in our planned development of the promising compounds coming out of this study.

12. Finally the authors should check the language once again.

The language has been thoroughly rechecked.

**Reviewer 1** | 01 Dec 2020 | 10:55

#1

Is the English language of sufficient quality?
- No

Is the quality of the figures and tables satisfactory?
- Yes

Does the reference list cover the relevant literature adequately and in an unbiased manner?
- Yes

Are the statistical methods valid and correctly applied? (e.g. sample size, choice of test)
- Yes

Are the methods sufficiently documented to allow replication studies?
- Yes

Are the data underlying the study available in either the article, supplement, or deposited in a repository? (Sequence/expression data, protein/molecule characterizations, annotations, and taxonomy data are required to be deposited in public repositories prior to publication)
- Yes

Does the study adhere to ethical standards including ethics committee approval and consent procedure?
- Yes

Have standard biosecurity and institutional safety procedures been adhered to?
- Not Applicable

**Reviewer 2:**

**Reviewer 2** | 06 Dec 2020 | 18:45

#1

A series of naphthalimide (NI) and naphthalene diimide (NDI)-based DNA binders are designed and DNA intercalations assessed through modelling and experimental work. Strong binding is observed in the molecular models but less conclusive in vitro. Anticancer activity is reasonable and better in the shorter chain systems than the longers ones.

Strengths:
- A computational and experimental approach has been adopted to investigate this novel series of DNA binding compounds
- The introduction is clear and motivations for performing the work are described well
- Figure 1 outlines the key molecular design principles
We profoundly thank the reviewer for this unbiased and scholarly assessment of our research piece. We have taken each and every comment very seriously and addressed them to the best of our present capability.

Limitations:
- A stronger case could be made for using NDI as the central aromatic unit instead of Ni (since this is a novel aspect of the work)

The present research piece reports the design of bisnaphthalimides with NDI as the linker and has been pictorial represented in figure 1. Taking into the suggestion of the reviewer, the appropriate changes have been done (in red font) to further highlight NDI portion of the designed chemical templates.

- It feels as if the computational and experimental work are presented as separate studies. The authors could do more to bring these components together, highlighting where conclusions are supported from multiple sources.

We thank the reviewer for pointing the need to bring out the conclusions from both computational and experimental part of the manuscript. As suggested, we have made appropriate additions that are highlighted in red font.

- The 'contributions to the field' could be improved. Currently, it is too long, detailed and technical. As a reader, we need to know why these molecules are important and what this work has uncovered in terms of designing anti-cancer drugs based on DNA binding.

As suggested by the reviewer, we have made appropriate changes in the manuscript which are highlighted in red font.

On page 8-10 all discussions of the DNA binding relates only to hydrogen bonding. All other non-covalent interactions that might drive intercalation i.e. aromatic stacking and hydrophobic forces are not discussed. Considering a key novelty of this work is the use of the aromatic NI and NDI motifs (and not just a hydrogen bond donating chain) I believe comparisons of aromatic stacking efficiency would also be important to make here.

We are grateful to the reviewer for raising this important aspect, which we missed. As suggested by the reviewer, we have incorporated other non-covalent interactions like hydrophobic interactions, pi-pi, pi-alkyl interaction in Table 1 and 2. Moreover, the detailed information on this is provided in the supplementary file and is appropriately cited in the text.

- In Tables 1 and 2, the labelling for locations of hydrogen bonds is difficult to interpret from just the Figures. Would it be possible to come up with a more general naming system with more clarity?
We thank the reviewer for pointing this out. The naming and numbering are generated by default in the AutoDock program. However, to address this issue we have generated the 2D structures of the most promising DNA interactive compounds. The 2D figures are provided in the supplementary file and this will be helpful to unambiguously understand where the ligand-DNA interactions are taking place.

- I would like to see 1H NMR spectra of all compounds included, not just peaks listed.
We have provided the characterization data of selected compounds in the supplementary information.

- Also NMR have been recorded in CDCl3 indeed suggesting these are relatively hydrophobic molecules (particularly shorter chain 5a,b). The aqueous solubility of these molecules should be examined and aggregation charcaterised e.g. DLS. It might be that the superior behaviour of the shorter chain 5a,b compounds might be related to their greater susceptibility to aggregate because they will be more hydrophobic. This should be considered.

We deeply thank the reviewer for directing us to a new path that will be helpful to understand the superior behavior of the shorter chain chemical templates. Unfortunately, because of the constraints resulting from this prevailing pandemic conditions, we could not take this up now. Moreover, aggregation characterization instruments are unavailable and needs research collaboration. We will definitely work on this in our future plans. We hope the reviewer will understand our plight.

- I think it might be important to discuss the limitations of the molecular models used. For example, solvent is not discussed. Are these molecular dynamics simulations performed in a solvent box? In which case this might explain why there are some discrepancies between the strength of binding reported by the models and found experimentally (i.e. in solution studies).

We thank the reviewer for this observation. In the present study, we have focused and performed molecular interaction analysis using AutoDock by removing HETATOM and water molecules from the native PDB 3D structure files. These analyses provide a good account of the possible interactions between the ligand-DNA and helpful in design of new chemical compounds. The molecular dynamics simulations indeed help to further validate and understand the results unambiguously, however these studies were not taken up as our focus shifted to evaluate the compounds experimentally. We will take this up in our future course of action with the promising molecules.

- Please add yields to Figure 4 (which is in fact a Scheme)

As suggested by the reviewer, the yields are added and the caption is changed as “scheme 1”
rather than “figure 4”

- I am not sure why the in silico studies (Table 1 and 2) make comparisons to compounds 3 and 4 but then the thermal denaturation studies make comparisons with DC-81. It would be more helpful to use the same controls throughout so that comparisons drawn in both are the same.
The DC-81 is one of the most common standards used in thermal denaturation studies with numerous literature precedence and was available to us. The comparison of the results with the compounds 3 and 4 in the prevailing conditions will be difficult now and we request the reviewer to consider our plight.

- In Tables 1 and 2, I do not think it necessary to provide the HB bong lengths with such accuracy. What are the errors here based on the molecular models? It is hard to pull out trends with numbers quoted to 5dp
As suggested by the reviewers, we have provided the hydrogen bonds lengths in 2 decimal points only. There were no errors, earlier we put the results as obtained, which were in 5 dp.

- I am concerned about the delta Tm values in Table 3. The errors on these are all +/- 0.2 deg C. Therefore, I would say that there has to be a > 0.4 deg C difference between Tm values in order to make a meaningful comparison with DC-81. This is not true for any of the values. Please can the authors rationalise why comparisons are valid when the errors in these calculated values are high.

We thank the reviewer for pointing this and we regret for the misleading typographical error. Similar to Tm values of 69.2 °C ± 0.01, all the ∆*T*m values are ± 0.01- 0.02°C and this has been corrected in the manuscript.

Is the English language of sufficient quality?
- Yes

Is the quality of the figures and tables satisfactory?
- No

Does the reference list cover the relevant literature adequately and in an unbiased manner?
- Yes

Are the statistical methods valid and correctly applied? (e.g. sample size, choice of test)
- No

Are the methods sufficiently documented to allow replication studies?
- Yes

Are the data underlying the study available in either the article, supplement, or deposited in a repository? (Sequence/expression data, protein/molecule characterizations, annotations, and taxonomy data are required to be deposited in public repositories prior to publication)
- No

Does the study adhere to ethical standards including ethics committee approval and consent procedure?
- Yes

Have standard biosecurity and institutional safety procedures been adhered to?
- Yes

In box 3 above I have detailed the key issues with the scientific conclusions of this work that should be addressed. These major points are summarised below:
- Amend the ‘contributions to the field’ summary to make it more accessible to non-experts in DNA binding
- Highlight which conclusions are supported from data provided from both modelling and experimental work
- Review the modelling data in Tables 1 and 2 for role of aromatic stacking interactions in dictating binding strength, i.e. the role of the NI and NDI motifs that present important novelty to this work. Use a clearer naming system to identify the hydrogen bonds shown in associated figures.
- Perform experiments on aggregation of these compounds under aqueous conditions (e.g. DLS) and discuss the implications for activity (i.e. shorter chain systems less soluble?)
- Discuss the limitations of the molecular modelling i.e. gas phase vs solution.
- Reconsider drawing comparisons between delta Tm values (Table 3) when errors are +/- 0.2 deg C.
- In the penultimate line of the conclusions, please clarify what is meant by ‘the low cytotoxicity of the compounds could be attributed to decreased bioavailability’ by referring specifically to the chemical structure of the novel class of DNA binders presented here (addressed appropriately)
